# The Phylogeography of Y-Chromosome Haplogroup H1a1a-M82 Reveals the Likely Indian Origin of the European Romani Populations

**DOI:** 10.1371/journal.pone.0048477

**Published:** 2012-11-28

**Authors:** Niraj Rai, Gyaneshwer Chaubey, Rakesh Tamang, Ajai Kumar Pathak, Vipin Kumar Singh, Monika Karmin, Manvendra Singh, Deepa Selvi Rani, Sharath Anugula, Brijesh Kumar Yadav, Ashish Singh, Ramkumar Srinivasagan, Anita Yadav, Manju Kashyap, Sapna Narvariya, Alla G. Reddy, George van Driem, Peter A. Underhill, Richard Villems, Toomas Kivisild, Lalji Singh, Kumarasamy Thangaraj

**Affiliations:** 1 CSIR-Centre for Cellular and Molecular Biology, Uppal Road, Hyderabad, India; 2 Evolutionary Biology Group, Estonian Biocentre, Tartu, Estonia; 3 Department of Evolutionary Biology, Institute of Molecular and Cell Biology, University of Tartu, Tartu, Estonia; 4 Himalayan Languages Project, Institut für Sprachwissenschaft, Universität Bern, Bern, Switzerland; 5 Department of Genetics, Stanford University School of Medicine, Stanford, California, United States of America; 6 Estonian Academy of Sciences, Tallinn, Estonia; 7 Department of Biological Anthropology, University of Cambridge, Cambridge, United Kingdom; 8 Genome Foundation, Hyderabad, India; 9 Banaras Hindu University, Varanasi, India; Kunming Institute of Zoology, Chinese Academy of Sciences, China

## Abstract

Linguistic and genetic studies on Roma populations inhabited in Europe have unequivocally traced these populations to the Indian subcontinent. However, the exact parental population group and time of the out-of-India dispersal have remained disputed. In the absence of archaeological records and with only scanty historical documentation of the Roma, comparative linguistic studies were the first to identify their Indian origin. Recently, molecular studies on the basis of disease-causing mutations and haploid DNA markers (i.e. mtDNA and Y-chromosome) supported the linguistic view. The presence of Indian-specific Y-chromosome haplogroup H1a1a-M82 and mtDNA haplogroups M5a1, M18 and M35b among Roma has corroborated that their South Asian origins and later admixture with Near Eastern and European populations. However, previous studies have left unanswered questions about the exact parental population groups in South Asia. Here we present a detailed phylogeographical study of Y-chromosomal haplogroup H1a1a-M82 in a data set of more than 10,000 global samples to discern a more precise ancestral source of European Romani populations. The phylogeographical patterns and diversity estimates indicate an early origin of this haplogroup in the Indian subcontinent and its further expansion to other regions. Tellingly, the short tandem repeat (STR) based network of H1a1a-M82 lineages displayed the closest connection of Romani haplotypes with the traditional scheduled caste and scheduled tribe population groups of northwestern India.

## Introduction

The Roma in England are traditionally known as gypsies because it was thought that they came from Egypt and were therefore ‘*gypcians’*. German *Zigeuner*, French *tzigane* and names in several other European languages derive from a designation for a Manichaean sect that practiced sorcery and soothsaying in the last centuries of the Byzantine Empire. On linguistic grounds, Grellmann [1] pointed out that the Roma must have originated in the Indian subcontinent. A detailed linguistic study by Pott [2] established that the various dialects spoken by the Roma derive specifically from North India. Roma populations are distributed widely within Europe including the Balkans and Scandinavia as well as throughout the Near East.

The name by which Roma designate themselves is *Rroma* (singular *Rrom*), whereby the double rr in Romani orthography represents a uvular ‘r’ [R] as opposed to an apical ‘r’ [r]. The autonym *Rroma* is held to be cognate with 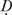 *oma*, a collective term for the ancient aboriginal populations of the Indian subcontinent. Many 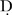
oma remained outcastes or tribals, whereas some were assimilated into the lower strata of the caste system by the Indo-European speaking Indians [3], [4].

The Roma route of migration has long been the object of linguistic study. Grierson [5] propagated the idea that the ‘Gipsy languages’ were of ‘Dardic origin’, but Turner [6] demonstrated that the Romani languages were not Dardic, but belonged to the same central Indo-Aryan subgroup as Hindi. The presence of Burushaski loans in Romani [7], the lack of Arabic loans and the presence of Dardic, Georgian, Ossetian, Armenian and mediaeval Greek loans [8] indicate that the Roma migrated to Europe by a northerly route, beginning around Gilgit in the northernmost Hindu Kush, thence along the southern Caspian littoral, the southern flank of the Caucasus, the southern shoreline of the Black Sea, across the Bosporus, and subsequently spreading across Europe since 13^th^ century. A legacy of the migration is that some Roma refer to themselves as Sinti, an adjectival form derived from Sindh, the name of the Indus river. Morgenstierne [9] argued that the endangered 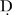
omākī language spoken by several hundred 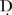
oma in Gilgit and Yasin, belonging to the minstrel and blacksmith castes, represents an ethnolinguistic remnant of the early Roma migration through what today is northern Pakistan.

The Indian ancestry of Roma has been studied using mtDNA, Y-chromosomal and autosomal studies [10]–[16]. The South Asian-specific mtDNA haplogroups M5a1, M18 and M35b [13], [16], single Y-chromosomal haplogroup (hg) H1a1a-M82 [10], [17] and the pathogenic 1267delG mutation in *CHRNE*, i.e. cholinergic receptor, nicotinic, epsilon [18], root Roma ancestry in South Asia. It is also evident that the Roma exchanged a significant amount of genes with contemporary populations on their way to Europe [16], [17] (and references therein). The latter finding corroborates the admixture *en route* explicitly proposed by linguists in the 19^th^ century [2], [19]. The genetic analysis of European Roma populations identified them as a suitable founder population to study Mendelian disorder and a valuable part of the European genetic landscape [12], [14], [15], [20], [21]. Recently, a study on primary congenital glaucoma reported a common mutation in the Roma and Jatt populations [21].

Although the Indian origin of the European Roma populations is linguistically and genetically well-established, accurate identification of their South Asian source has remained a matter of debate. Some linguistic studies argue that the proto-Romani founder population must have been in northwestern India [22]–[24], although their own origin myth suggests an origin on the Gangetic plain [25], [26]. The classical and mtDNA genetic markers suggested the closest affinity of the Roma with Rajput and Punjabi populations from Rajasthan and the Punjab respectively [16], [20], although these studies were compromised by low level phylogenetic resolution and a limited coverage of the Indian populations. These findings are now reconciled in light of our present genetic study thoroughly, comparing paternal lineages of Roma and a large number of South Asian populations, throwing light on the relationships and recent split of these groups.

In contrast with the maternal lineages, where three South Asian-specific lineages were reported [16], the paternal lineage of Roma carries only a single currently discernible South Asian-specific Y-chromosomal founder hg H1a1a-M82, ranging from 10 to 60% frequency in various Roma populations [10], [17], [27]–[31]. The paternal ancestor of hg H1a1a-M82, viz. hg H-M69, is an autochthonous component of the South Asian Y-chromosome gene pool, accounting, on average, for 22% of the studied paternal lineage pools [32]–[36]. Its relatively deep phylogenetic position within the overall Y-chromosome phylogeny as well as its extremely low frequency elsewhere, including the East and Southeast Asia, Middle East and Europe [32], [37]–[43], suggests that it arose in South Asia sometime prior to the Last Glacial Maximum, perhaps even earlier between 30 and 40 KYA ago [33].

There are examples where a single Y-chromosomal hg is associated with the expansion of a population from a homeland, e.g. hg C3 with Genghis Khan's army [44], spread of hg O1-M110 bearing Austronesians from Taiwan to the Admiralty Islands of Melanesia [45] and hg O2a-M95 with the incursion of Austroasiatic speakers into India [35]. The reduced diversity and expansion of H1a1a-M82 lineages in all Roma groups argues for a descent from a single paternal ancestor in the Indian subcontinent [10], [29]. The most popular model proposes an origin in northwestern India, but this model has not been tested using an adequately large collection of data from Y-chromosomes of the Indian subcontinent. Here, we address this issue by generating the largest dataset to date of H1a1a-M82 lineages including 214 ethnic populations from India that fill a major geographical gap (Table S1).

To detect possible source populations of European Roma paternal lineages within, we genotyped first M82-H1a1a hg) in a large number of Indian populations to produce a high-resolution dataset of hg H1a1a-M82 and thereafter, 17 Y-chromosome short tandem repeats (STRs) (Text S1 and Table S2). To reduce sampling bias, we have analysed more than 7000 samples from 214 ethnic populations covering the whole length and breadth of India (Table S1 and S3).

## Results and Discussion

**Figure 1:**
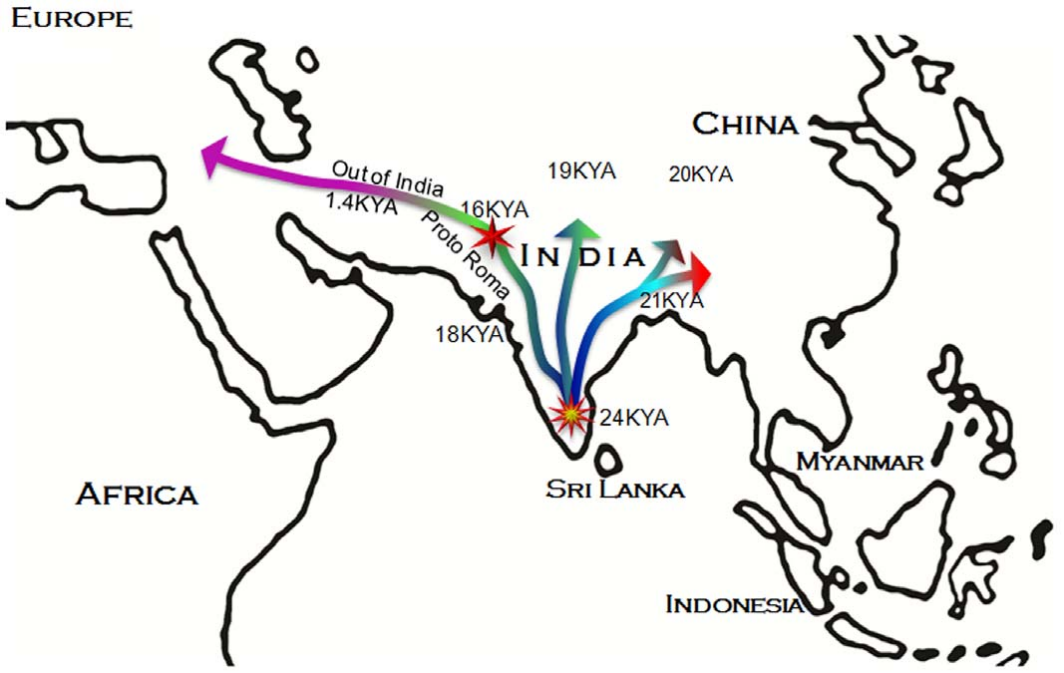
The most parsimonious route of prehistoric expansion of Y-chromosomal haplogroup H1a1a-M82 and the recent out-of -India migration of European Roma ancestors.

Hg H1a1a-M82 accounts for more than 12% of all male lineages in South Asia, with a highest incidence of 20% amongst South Indians and a lowest incidence of 0.2% among northeast Indian populations (Table S1 and S3). Hg H1a1a-M82 has a decreasing gradient from its peak frequency in southern India toward both the northwestern and the northeastern peripheries and a virtual absence further to the extreme east and west (Table S1, S3 and S4). Interestingly, substantial frequency changes occur despite the short geographical distance between the East Indian and their adjacent northeast Indian populations, suggesting different paternal population histories for these two neighboring regions. Consistent with the frequency distribution of H1a1a-M82 within India, the Y-STR variance and expansion ages basically reflect a similar pattern (Fig. 1 and Table 1). The overall TMRCA estimate of the H1a1a-M82 lineage in India using ρ-statistics [46], was ∼22KYA. The regional expansion times within India range between 16 and 24 KYA (Table 1). The higher expansion time with the associated mean pair-wise difference (MPD) and haplotype diversity (HD) (Fig. 1 and, Table 1 and 2) suggests that hg H1a1a-M82 originated in the same South Asian pre-LGM gene pool of Y-chromosomes.

**Table 1:** Measure of Genetic Diversity and expansion ages of haplogroup H1a1a-M82 among different population groups.

Group	*n*	*h*	HD	MPD	AMD	Variance	Age
Roma Portugal	21	8	0.8286±0.0646	1.5190±0.9501	1.14	0.08	3.69±2.53
Roma Serbia	41	17	0.8744±0.0363	1.4183±0.8851	1.66	0.1	3.71±2.18
Roma Croatia	76	13	0.5607±0.0674	1.2758±0.8111	1.36	0.05	1.91±0.72
Northwest India	64	57	0.9950±0.0042	7.0164±3.3392	5.86	0.38	16.04±2.38
West India	13	13	1.0000±0.0302	6.6795±3.3706	5.23	0.45	17.65±3.97
North India	26	24	0.9908±0.0151	8.3108±3.9781	8.04	0.49	19.46±2.95
East India	14	13	0.9890±0.0314	8.0659±3.9862	9.14	0.48	20.79±3.84
Northcentral India	35	20	0.9092±0.0389	7.3126±3.5062	7.94	0.46	19.12±3.01
Southcentral India	43	36	0.9900±0.0078	8.7741±4.1281	8.84	0.59	24.94±3.09
South India	37	35	0.9970±0.0071	8.8769±4.1865	8.78	0.56	23.63±3.22

*n* = number of samples; *h* = number of haplotypes; HD = haplotype diversity; MPD = mean pairwise difference; AMD = average mutational distance from Roma modal haplotype.

**Table 2:** The average number of pairwise differences are shown within (PiX along diagonal) and between (PiXY above diagonal) populations; pairwise (δμ)2 genetic distance values are depicted below the diagonal.

	RP	RS	RC	NWI	WI	NI	EI	NCI	SCI	SI	Afg
Roma-Portugal (RP)	1.51905	1.73984	2.14662	5.52827	4.7326	7.39011	8.26871	7.02449	7.49723	7.65508	6.34921
Roma-Serbia (RS)	0.27117	1.41829	2.42202	5.75191	4.88368	7.59568	8.40592	7.06899	7.64265	7.61437	6.61382
Roma-Croatia (RC)	0.7492	1.07497	1.27579	6.03372	4.90891	7.92814	7.98872	7.22744	7.54376	7.98898	6.02851
Northwest India (NWI)	1.26057	1.53457	1.88764	7.01637	7.0012	8.0631	8.66853	8.08304	8.45858	8.46917	7.52344
West India (WI)	0.63333	0.83479	0.93127	0.15327	6.67949	8.24852	8.47802	7.8044	8.19678	8.35135	7.17949
North India (NI)	2.4752	2.73115	3.13486	0.39953	0.75339	8.31077	9.12363	8.14945	8.85599	8.79002	8.19231
East India (EI)	3.47622	3.66381	3.31786	1.12738	1.10531	0.93527	8.06593	8.62653	9.26578	8.94402	7.4881
Northcentral India (NCI)	2.60866	2.70354	2.93325	0.91855	0.80835	0.33776	0.93726	7.31261	8.6	8.60541	7.72857
Southcentral India (SCI)	2.35066	2.54647	2.51882	0.56335	0.46999	0.31357	0.84577	0.55665	8.77409	9.04274	8.18605
South India (SI)	2.45712	2.46679	2.91264	0.52255	0.57317	0.1962	0.47261	0.51066	0.21726	8.87688	8.27928
Afghani (Afg)	2.92302	3.23801	2.72395	1.34859	1.17308	1.37026	0.78846	1.4056	1.13234	1.17417	5.33333

Within India, language group-wise expansion times and variance were observed to be highest among Dravidian speakers (25±3.5 KYA, 0.59), followed by Austroasiatic speakers (22.4±3.3 KYA, 0.52), and lowest among Indo-European speakers (19.5±2.2 KYA, 0.47). Just as with the expansion time of hg O2a-M95 among Austroasiatic speakers [35], the H1a1a-M82 expansion time was established to be substantially higher among South Munda (22±3.5 KYA) than in the North Munda (16.3±3 KYA) group. The South Munda groups live in close proximity to the Dravidian groups, and it is likely that the already diverse Dravidian H1a1a-M82 might have assimilated into the Munda group after the Austroasiatic incursion into the Indian subcontinent.

**Figure 2:**
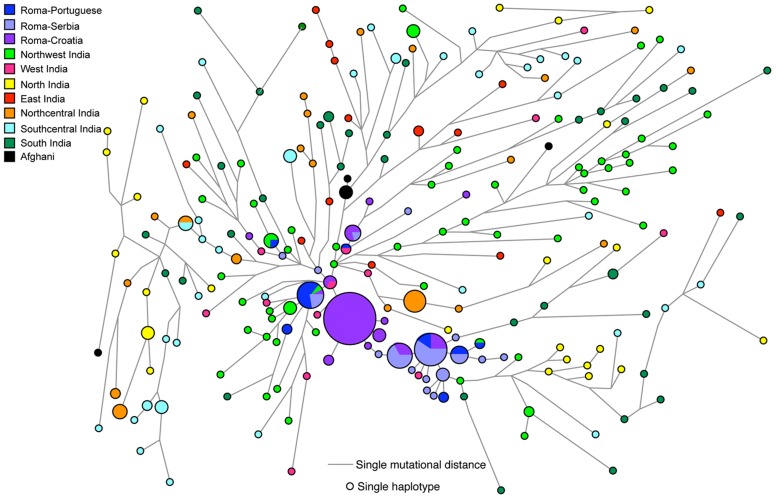
Phylogenetic network relating Y-STR haplotypes within haplogroup H1a1a -M82. The network was constructed using a median joining with MP (maximum parsimony) algorithm as implemented in the Network 4.6 program. The size of the circles is proportional to the number of samples. The data used for comparison have been taken from the literature (See Text S1).

The geographical distribution of hg H1a1a-M82 is largely restricted to South Asia, and its significant occurrence among European Roma populations strongly links the Roma to the Indian subcontinent. The high frequency of H1a1a-M82 among all the Roma groups and their reduced genetic diversity relative to South Asian populations can most likely be attributed to their recent migration from India (Table 1 and 2). In the network analysis (Fig. 2), the Roma Y-STR haplotypes cluster predominantly close to the northwestern Indian haplotypes. In addition, the northwestern Indian haplotypes, while diverse, generally radiate from the core of the network while the Roma haplotypes being distributed further away. These patterns point to northwestern India as the source of the Roma H1a1a-M82 chromosomes. The average age estimate of Roma founders considering their distance from Northwest Indian founders is 1405±688 YBP (Table 3), which is largely in agreement with the time frames suggested previously.

**Table 3:** Y-chromosomal haplogroup H1a1a-M82 founder analysis for Roma.

*n* (number of samples) in the cluster	TMRCA (years)	SD (years)
5	1110	785
65	1024	407
61	2081	873
	**1405**	**688**

Different founders were identified based on the Network analysis (Text S1). The age was estimated from the ρ statistic (the mean number of mutations from the assumed root of each and every founder), using a 25-year generation time and the TD statistic, assuming a mutation rate of 6.9×10^−4^ [11], based on variation at 15 common Y-STR loci.

**Figure 3:**
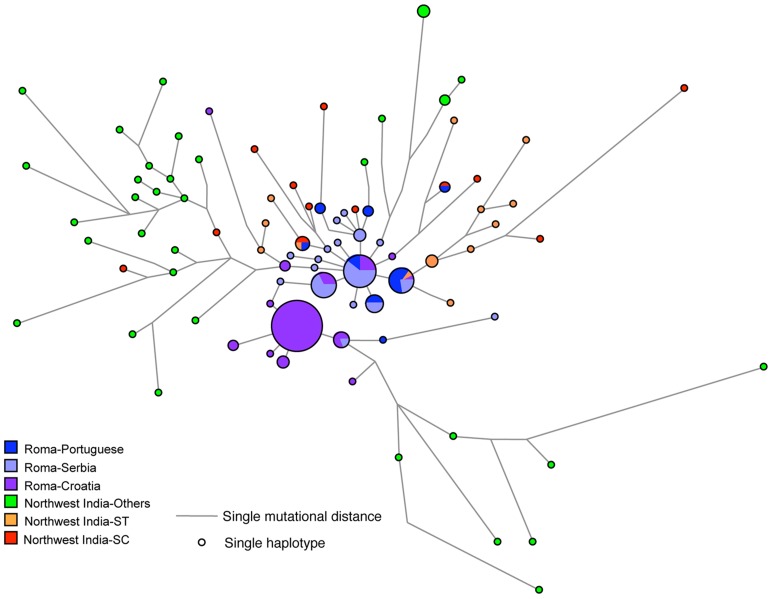
Phylogenetic network relating Y-STR haplotypes within haplogroup H1a1a -M82- of Roma with northwest Indian populations. The network was constructed using a median joining with MP (maximum parsimony) algorithm as implemented in the Network 4.6 program. The size of the circles is proportional to the number of samples. SC = Scheduled Caste and ST = Scheduled Tribe.

In order to ascertain the closest population group among northwestern Indians, we redrew the network of Roma haplotypes exclusively within the northwestern Indian variation (Fig. 3). It is highly revealing that the closest or matching haplotypes with the Roma haplotypes were found in scheduled caste and scheduled tribe populations, while the middle and upper caste haplotypes were more distant to the Roma haplotypes (Fig. 3). Scheduled castes and Scheduled tribes are the endogamous groups in India that are given a special status by the Government of India to uplift their social status (for more details, refer [47]). Historically, the assimilation of so-called tribals into the caste system generally did little to ameliorate the socio-economic barriers or enhance the marriageability of former outcastes to members of the middle or high castes. However their language and means of subsistence were often affected, e.g. assimilation to an Indo-Aryan language and the shift from foraging, hunting and fishing to a more sedentary existence. Not surprisingly, the genetic differences between scheduled tribes and scheduled castes are not found to be substantial [47]. On the basis of our findings, it is therefore most parsimonious to conclude that the genealogically closest patrilineal ancestors of the Roma were among the ancestors of the present scheduled tribes and scheduled caste populations of northwestern India. The genetic data analysed here for the first time provide strong population genetic support for the linguistic based identification of the ancestral Roma with the presumed aboriginal 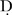
oma of northwestern India and the Gangetic plain.

This suggestion also enables a resolution of the controversy on the Punjab and the Gangetic plain. The 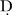
oma are indigenous to the Punjab as well as to the entire Gangetic plain as far east as the lower course of the Brahmaputra. Yet not all 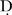
oma groups were historically ancestral to the European Roma, and in fact most descendants of the aboriginal 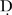
oma reside in the Indian subcontinent today [4]. It is held that a large number of aboriginal 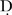
oma were recruited in the Punjab to repel the Ghaznavid invasions of the kingdom of Jayapāla between 1001 and 1026. These 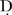
oma were rewarded by nominal promotion to K
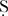
atriya ‘warrior’ or Rājpūt caste status, but such nominal promotions in the social context of the Indian subcontinent generally did not lead to a genuine enhancement of eligibility in marriage or social status because of the enduring nature of local memory [4]. With the fall of the Hindu polities in what today is Pakistan, the westward migration of the 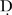
oma was set into motion. Arguably, the humble social position of the 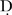
oma could frequently have led to circumstances, which could have promoted their geographical mobility. Yet the timing of the Ghaznavid invasion presents the probable temporal and spatial historical correlation.

In order to conduct an intra-group comparison of Y-chromosome variation, we estimated the genetic distances (RST values) from 15 loci Y-STR haplotypes and made a multidimensional scaling plot (Fig. S1). The first dimension largely separated all the Indian groups from one another, while the second dimension had a maximum impact in separating east Indian from north-central Indian and northwestern Indian from the Roma groups as well as Croatian Roma from Portuguese and Serbian Roma. We also noted other significant genetic differences between Croatian Roma and Portuguese and Serbian Roma. Portuguese and Serbian Roma share a closer affinity than Croatian Roma (Fig. S1 and Table S5). South and south-central Indian groups occupy a pivotal position in the plot, with other groups scattered around them in accordance with their geographical affinity. The modal haplotypes of different population groups are given in Table S6 and their distance to it in Table S7. The Analysis of Molecular Variance (AMOVA) based on Y-STR data shows that the Roma populations are closest to northwestern Indian populations (Table 4). The average mutational distance from the Roma modal haplotype shows a consistent pattern of northwestern Indian populations as representing the closest Indian groups to European Roma (Table S7).

**Table 4:** Analysis of Molecular Variance (AMOVA) using Y-STRs between groups of populations categorized on the basis of geography.

Group	Fst
Roma Vs. Northwest India	0.34990
Roma Vs. West India	0.36792
Roma Vs. North India	0.55269
Roma Vs. East India	0.65300
Roma Vs. Northcentral India	0.52934
Roma Vs. Southcentral India	0.45002
Roma Vs. South India	0.48570
Roma Vs. Afghani	0.68314

In conclusion, the analysis of Y-chromosome hg H1a1a-M82 variation in 214 ethnic groups from India shows that northwest Indian populations are the closest to the hg H1a1a-M82 variants observed in the present-day European Roma populations. Although only entire genome analyses can provide the richest narrative of history and migration, the Y-chromosome hg H1a provides an exceptional record of Indian and European Roma-specific paternal heritage, including their exodus from northwestern India and subsequent recent expansion. Our phylogeographical study of hg H1a1a-M82 enables now to better understand the temporal and spatial parameters of this migration. This first genetic evidence of this nature allows us to develop a more detailed picture of the paternal genetic history of European Roma, revealing that the ancestors of present scheduled tribes and scheduled caste populations of northern India, traditionally referred to collectively as the 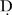
oma, are the likely ancestral populations of modern European Roma. Our findings corroborate the hypothesized cognacy of the terms *Rroma* and 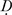 *oma* and resolve the controversy about the Gangetic plain and the Punjab in favour of the northwestern portion of the diffuse widespread range of the 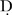
oma ancestral population of northern India.

## Materials and Methods

Samples were collected with the informed written consent from 3498 unrelated healthy individuals belonging to 57 populations from all the four linguistic groups of India (Table S1 and S3). Y-chromosome marker M82 (defining hg H1a) was genotyped in all the samples. From one to five M82 derived samples of each population were randomly selected for Y-STR genotyping based upon the frequency of H1a1a-M82 in the respective population. Our main aim was to cover highest geographic area (Table S1 and S3). In total, 204, M82 derived samples were genotyped by using the AmpFℓSTR® Y-filer™ PCR amplification Kit (Applied Biosystems). The detailed material and method section is given in Text S1.

## Supporting Information

Text S1 **Detailed** **materials and methods** **.**
(DOC)

Figure S1 **Multidimensional scaling plot of the Rst distances of the population groups based on haplogroup H1a1a-M82 Y-STR data.**
(TIF)

Table S1 **Details of the Indian and Nepali samples included in the present study and haplogroup H1a1a-M82 frequencies.**
(DOC)

Table S2 **The 15 loci Y-STR profile of haplogroup H1a1a-M82 belonging to Indian, Afghani and European Roma population, used in the present analysis.**
(DOC)

Table S3 **Regionwise haplogroup frequency in India and Nepal.**
(DOC)

Table S4 **The frequency of haplogroup H1a1a-M82 among different world populations.**
(DOC)

Table S5 **Mean pairwise Fst between different studied groups for haplogroup H1a1a-M82.**
(DOC)

Table S6 **Modal H1a1a-M82 Y-STR haplotype of different population groups.**
(DOC)

Table S7 **Average mutational distances from Roma Modal haplotype.**
(DOC)
