# Supplementary material for: The Phylogeography of Y-Chromosome Haplogroup H1a1a-M82 Reveals the Likely Indian Origin of the European Romani Populations
Source: PLoS One. 2012 Nov 28;7(11):e48477. doi: 10.1371/journal.pone.0048477 (PMC3509117; doi:10.1371/journal.pone.0048477)
Supplement: Text S1 — Detailed materials and methods . (DOC) [file pone.0048477.s001.doc]

**Materials and Methods**

***Sampling and genotyping:***

Blood or buccal swab samples were collected with the informed written consent from 3498 unrelated healthy individuals belonging to 57 populations from all the four linguistic groups of India (Supplementary Table S1 and S2). This project was approved by Institutional Ethical Committee (EIC) of CCMB and EBC. DNA isolation was performed using the standard protocol published elsewhere [[1]](http://wizfolio.com/?citation=1&ver=3&ItemID=3215&UserID=2886&AccessCode=0&CitationSuffix=). Typing haplogroup H1a1a specific Y-chromosomal marker M82 among 3498 individuals resulted, 409 individuals carrying this mutation. To amplify the template DNA using 10 pM of each primer, 100 µM dNTPs, 1.5 mM MgCl2 and 1 U of Taq DNA polymerase. Thirty five cycles of reaction were performed with 30 seconds denaturation at 94°C, 30 seconds annealing at 55°C and 2 minutes extension at 72°C. The final extension was done for 7 minute. Sequencing reaction was carried out with BigDye™ Terminator cycle sequencing kit (Applied Biosystems, USA) using ABI 3730XL DNA Analyzer (Applied Biosystems, USA). We compared our results with the published 4188 Y chromosomes belonging to 157 different populations from India subcontinent [[2](http://wizfolio.com/?citation=1&ver=3&ItemID=359&UserID=2886&AccessCode=78B3B0DCED384E07863A06203BD1064C&CitationSuffix=) [-13]](http://wizfolio.com/?citation=1&ver=3&ItemID=3362&UserID=2886&AccessCode=828CCB32BAFC412B8C9AC28A4A10BC49&CitationSuffix=) (Supplementary Table S1 and S4).

***Y-STR typing***

From one to five samples of each population derived to H1a1a-M82 marker were randomly selected for Y-STR genotyping based upon the frequency of H1a1a-M82 in the respective population. Our main aim was to cover highest geographic area (Figure 1). In total, 204, M82 derived samples were typed for loci DYS19, DYS389I and II, DYS385, DYS390, DYS391, DYS392, DYS393, DYS456, DYS458, DYS437, DYS438, DYS439, DYS448, DYS635 and Y GATA H4 by using the AmpFℓSTR® Y-filer™ PCR amplification Kit (Applied Biosystems) following the conditions (1) 95°C for 11 min, (2) 30 cycles: 94°C for 1 min, 61°C for 1 min, 72°C for 1 min, (3) 60°C for 80 min, and (4) 25°C hold. The PCR amplicons along with GS500 LIZ (as size standard) were run in the ABI 3730XL DNA Analyzer (Applied Biosystems, Foster City, USA). Fragment sizes were determined using the GeneMapper® Analysis Software v4.0 and allele designations were based on comparison with allelic ladders included in the Yfiler™ kit. The alleles were noted for all except changes in the two loci where “DYS389I” was used as “DYS389cd” and “DY389ab” = (DYS389II-DYS389I). Out of 17 loci obtained, two DYS385 loci were excluded from the current analyses because they could not be distinguished using the typing method employed. Thus, all the analysis linked with Y-STR data were carried out with 15 loci [[9,](http://wizfolio.com/?citation=1&ver=3&ItemID=3345&UserID=2886&AccessCode=7B015C10300D475FAFBF5C2ECBD2D73D&CitationSuffix=)[12,](http://wizfolio.com/?citation=1&ver=3&ItemID=3101&UserID=2886&AccessCode=35B3CB4A0120467EA029BA4EAE90A553&CitationSuffix=)[14](http://wizfolio.com/?citation=1&ver=3&ItemID=3344&UserID=2886&AccessCode=40709DA42F2842CE95590784B6E3E440&CitationSuffix=) [-17]](http://wizfolio.com/?citation=1&ver=3&ItemID=3332&UserID=2886&AccessCode=D708314671A94A5B923E21FE0088E611&CitationSuffix=) (Supplementary Table S3).

***Geographical division***

We divided geography of India in to seven different parts, and all the analysis were done based on following divisions-

Northwest India- Gujarat, Rajasthan, Haryana and Punjab

North India- Jammu and Kashmir, Himanchal Pradesh, Uttaranchal, Uttar Pradesh and Nepal

East India- Bihar, Jharkhand, West Bengal, Meghalaya and Orissa

Northcentral India- The north part of Vindhya Mountain in Madhya Pradesh

Southcentral India- The south part of Vindhya Mountain in Madhya Pradesh and Chhattishgarh

West India- Maharashtra

South India- Andhra Pradesh, Karnataka, Tamil-Nadu, Pondicherry and Kerala

The population names of individuals typed for Y-STR markers were not revealed to protect the donors to show their relationship with European Roma which they have not anticipated when they agreed their donation of blood/buccal swab for population genetics research.

***Statistical analysis***

Number of haplotypes, Fst distance, haplotype diversity, mean pairwise differences and analysis of molecular variance (Tables 1, 2 and 3 and Supplementary Table S5) of Y-STR for studied populations were calculated using the Arlequin 3.5 software package [[18]](http://wizfolio.com/?citation=1&ver=3&ItemID=2957&UserID=2886&AccessCode=AE408BEBE2E146268A419FDEA73E18E4&CitationSuffix=). Genetic distances (RST values) were calculated using 15 Y-STR loci and visualized with a multidimensional scaling plot in SPSS (Supplementary Figure S1). A median-joining network, resolved with the MP algorithm, was constructed using the (STR data presented in Supplementary Table 3), Network package (version 4.6) ([www.fluxus-engineering.com](http://www.fluxus-engineering.com/)); one Steiner tree is shown in figure 2 and 3. The age of M82-H1a1a was estimated from microsatellite variation within the haplogroup using the method described by [[19]](http://wizfolio.com/?citation=1&ver=3&ItemID=1082&UserID=2886&AccessCode=C3905B4CB0A344F5A16B23FDF4B3D682&CitationSuffix=) and updated in [[20]](http://wizfolio.com/?citation=1&ver=3&ItemID=1045&UserID=2886&AccessCode=C1987A98BF51472EA2A7B9894F64EA6B&CitationSuffix=). Moreover, different founders were identified based on Network analysis of Romani speakers, similar to the methodology published elsewhere [[10,](http://wizfolio.com/?citation=1&ver=3&ItemID=1431&UserID=2886&AccessCode=94077D8380834D22A51A639AEAC9F2FF&CitationSuffix=)[21](http://wizfolio.com/?citation=1&ver=3&ItemID=3252&UserID=2886&AccessCode=12316B846AF04490982BE10CC9683007&CitationSuffix=)[,22]](http://wizfolio.com/?citation=1&ver=3&ItemID=3358&UserID=2886&AccessCode=46AE17704ED84C3BA6FCBA4D0C5296E5&CitationSuffix=). The age of these founders (Table 4), was estimated from the ρ statistic (the mean number of mutations from the assumed root of each and every founder), using a 25-year generation time and the TD statistic, assuming a mutation rate of 6.9 × 10^−4^ [[19]](http://wizfolio.com/?citation=1&ver=3&ItemID=1082&UserID=2886&AccessCode=C3905B4CB0A344F5A16B23FDF4B3D682&CitationSuffix=), based on variation at 15 common Y-STR loci (Supplementary Table S3). To get the information about hypothetical most likely central haplotype close to the original founding lineage for each group, we have also calculated modal haplotype of different population groups and average mutational distances from Roma Modal haplotype in Microsoft excel (Supplementary Tables S6 and S7).

[**References**](https://wizfolio.com/?style=1&ver=3&UserID=2886&StyleName=PLoS One)

1. Thangaraj K, Joshi MB, Reddy AG, Gupta NJ, Chakravarty B et al. (2002) CAG repeat expansion in the androgen receptor gene is not associated with male infertility in Indian populations. J Androl 23: 815-818.

2. Thangaraj K, Singh L, Reddy AG, Rao VR, Sehgal SC et al. (2003) Genetic affinities of the Andaman Islanders, a vanishing human population. Curr Biol 13: 86-93.

3. Kivisild T, Rootsi S, Metspalu M, Mastana S, Kaldma K et al. (2003) The genetic heritage of the earliest settlers persists both in Indian tribal and caste populations. Am J Hum Genet 72: 313-332.

4. Zerjal T, Pandya A, Thangaraj K, Ling EYS, Kearley J et al. (2007) Y-chromosomal insights into the genetic impact of the caste system in India. Hum Genet 121: 137-144.

5. Reddy BM, Langstieh BT, Kumar V, Nagaraja T, Reddy ANS et al. (2007) Austro-Asiatic tribes of Northeast India provide hitherto missing genetic link between South and Southeast Asia. PLoS ONE 2: e1141.

6. Chaubey G, Metspalu M, Karmin M, Thangaraj K, Rootsi S et al. (2008) Language shift by indigenous population: a model genetic study in South Asia. Int J Hum Genet 8: 41.

7. Trivedi R, Sahoo S, Singh A, Bindu G, Banerjee J et al. (2008) Genetic imprints of Pleistocene origin of Indian populations: a comprehensive phylogeographic sketch of Indian Y-chromosomes. Int J Hum Genet 8: 97-118.

8. Fornarino S, Pala M, Battaglia V, Maranta R, Achilli A et al. (2009) Mitochondrial and Y-chromosome diversity of the Tharus (Nepal): a reservoir of genetic variation. BMC Evol Biol 9: 154.

9. Thangaraj K, Naidu BP, Crivellaro F, Tamang R, Upadhyay S et al. (2010) The influence of natural barriers in shaping the genetic structure of Maharashtra populations. PLoS ONE 5: e15283.

10. Chaubey G, Metspalu M, Choi Y, Mägi R, Romero IG et al. (2011) Population genetic structure in Indian Austroasiatic speakers: the role of landscape barriers and sex-specific admixture. Mol Biol Evol 28: 1013-1024.

11. Shah AM, Tamang R, Moorjani P, Rani DS, Govindaraj P et al. (2011) Indian siddis: African descendants with Indian admixture. Am J Hum Genet 89: 154-161.

12. Sharma G, Tamang R, Chaudhary R, Singh VK, Shah AM et al. (2012) Genetic affinities of the central Indian tribal populations. PLoS ONE 7: e32546.

13. Debnath M, Palanichamy MG, Mitra B, Jin JQ, Chaudhuri TK et al. (2011) Y-chromosome haplogroup diversity in the sub-Himalayan Terai and Duars populations of East India. J Hum Genet 56: 765-771.

14. Gusmão A, Gusmão L, Gomes V, Alves C, Calafell F et al. (2008) A perspective on the history of the Iberian Gypsies provided by phylogeographic analysis of Y-chromosome lineages. Ann Hum Genet 72: 215-227.

15. Klarić IM, Salihović MP, Lauc LB, Zhivotovsky LA, Rootsi S et al. (2009) Dissecting the molecular architecture and origin of Bayash Romani patrilineages: genetic influences from South-Asia and the Balkans. Am J Phys Anthropol 138: 333-342.

16. Regueiro M, Stanojevic A, Chennakrishnaiah S, Rivera L, Varljen T et al. (2011) Divergent patrilineal signals in three Roma populations. Am J Phys Anthropol 144: 80-91.

17. Lacau H, Gayden T, Regueiro M, Chennakrishnaiah S, Bukhari A et al. (2012) Afghanistan from a Y-chromosome perspective. Eur J Hum Genet 20:1063-1070.

18. Excoffier L, Laval G, Schneider S (2005) Arlequin (version 3.0): An integrated software package for population genetics data analysis. Evolutionary Bioinformatics Online 1: 47-50.

19. Zhivotovsky LA, Underhill PA, Cinnioglu C, Kayser M, Morar B et al. (2004) The effective mutation rate at Y chromosome short tandem repeats, with application to human population-divergence time. Am J Hum Genet 74: 50-61.

20. Sengupta S, Zhivotovsky LA, King R, Mehdi SQ, Edmonds CA et al. (2006) Polarity and temporality of high-resolution y-chromosome distributions in India identify both indigenous and exogenous expansions and reveal minor genetic influence of Central Asian pastoralists. Am J Hum Genet 78: 202-221.

21. Zerjal T, Xue Y, Bertorelle G, Wells RS, Bao W et al. (2003) The genetic legacy of the Mongols. Am J Hum Genet 72: 717-721.

22. Pemberton TJ, Li FY, Hanson EK, Mehta NU, Choi S et al. (2012) Impact of restricted marital practices on genetic variation in an endogamous Gujarati group. Am J Phys Anthropol. 149:92-103
