# Supplementary figures and images for: The Phylogeography of Y-Chromosome Haplogroup H1a1a-M82 Reveals the Likely Indian Origin of the European Romani Populations
Source: PLoS One. 2012 Nov 28;7(11):e48477. doi: 10.1371/journal.pone.0048477 (PMC3509117; doi:10.1371/journal.pone.0048477)

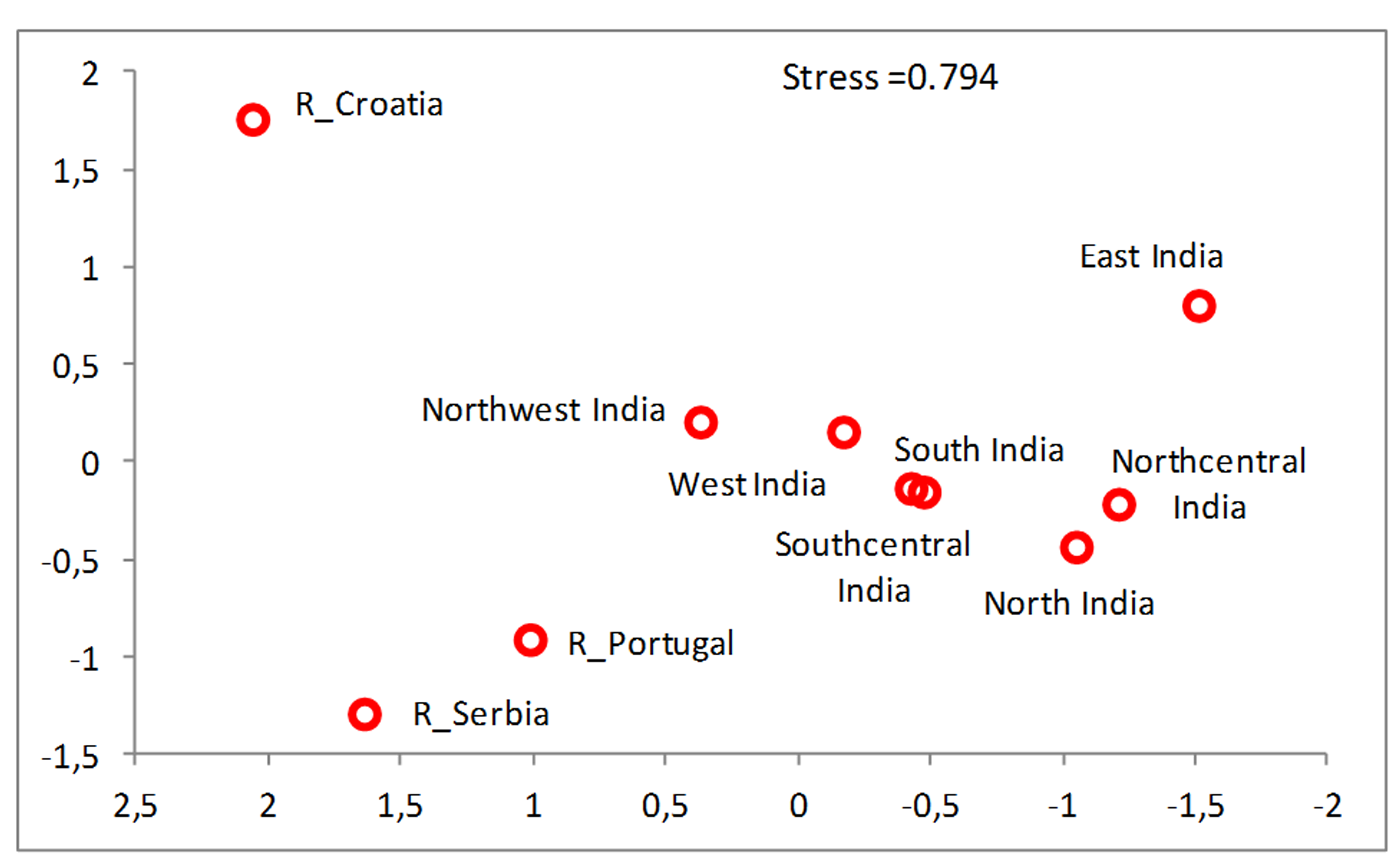

Supplement: Figure S1 — Multidimensional scaling plot of the Rst distances of the population groups based on haplogroup H1a1a-M82 Y-STR data. (TIF) [file pone.0048477.s002.tif]
