# Supplementary material for: The Phylogeography of Y-Chromosome Haplogroup H1a1a-M82 Reveals the Likely Indian Origin of the European Romani Populations
Source: PLoS One. 2012 Nov 28;7(11):e48477. doi: 10.1371/journal.pone.0048477 (PMC3509117; doi:10.1371/journal.pone.0048477)
Supplement: Table S1 — Details of the Indian and Nepali samples included in the present study and haplogroup H1a1a-M82 frequencies. (DOC) [file pone.0048477.s003.doc]

| **S.No.** | **Population** | **Region** | **State** | **Linguistic Affiliation** | **Total** | **Hg. H1a freq.** | **Reference** |
| --- | --- | --- | --- | --- | --- | --- | --- |
| 1 | Yadav | Northwest | Rajasthan | Indo-European | 96 | 0.031 | Present study |
| 2 | Dokali | South | Andhra Pradesh | Dravidian | 50 | 0.18 | Present study |
| 3 | Medari | South | Andhra Pradesh | Dravidian | 50 | 0.1 | Present study |
| 4 | Baniya | Northwest | Haryana | Indo-European | 43 | 0.093 | Present study |
| 5 | Scheduled Castes | Northwest | Haryana | Indo-European | 38 | 0.237 | Present study |
| 6 | Backward Castes | Northwest | Haryana | Indo-European | 41 | 0.098 | Present study |
| 7 | Pandit | Northwest | Haryana | Indo-European | 41 | 0.122 | Present study |
| 8 | Silawat | Central | Madhya Pradesh | Indo-European | 99 | 0.182 | Present study |
| 9 | Chenchu | South | Andhra Pradesh | Dravidian | 43 | 0.209 | Present study |
| 10 | Lambadi | South | Andhra Pradesh | Indo-European | 59 | 0.051 | Present study |
| 11 | Jatav | North | Uttar Pradesh | Indo-European | 32 | 0.094 | Present study |
| 12 | Kurmi | North | Uttar Pradesh | Indo-European | 42 | 0.381 | Present study |
| 13 | Konda Kammara | South | Andhra Pradesh | Dravidian | 77 | 0.234 | Present study |
| 14 | Santhal | East | Jharkhand | Austroasiatic | 99 | 0.152 | Present study |
| 15 | Darjeeling Gorkhas | East | West Bengal | Tibeto-Burman | 173 | 0.029 | Present study |
| 16 | Nyshi | Northeast | Arunachal-Pradesh | Tibeto-Burman | 48 | 0.021 | Present study |
| 17 | Bhootia | North | Uttarakhand | Tibeto-Burman | 55 | 0.018 | Present study |
| 18 | Tibetan Refugees | South | Karnataka | Tibeto-Burman | 109 | 0.009 | Present study |
| 19 | Muduliar | South | Andhra Pradesh | Dravidian | 21 | 0.143 | Present study |
| 20 | Oddari | South | Andhra Pradesh | Dravidian | 40 | 0.375 | Present study |
| 21 | Yarkali | South | Andhra Pradesh | Dravidian | 11 | 0.091 | Present study |
| 22 | Ladia | Central | Madhya Pradesh | Indo-European | 65 | 0.2 | Present study |
| 23 | Chaurasia | Central | Madhya Pradesh | Indo-European | 73 | 0.11 | Present study |
| 24 | Budagajangam | South | Andhra Pradesh | Indo-European | 96 | 0.146 | Present study |
| 25 | Patel | Northwest | Gujarat | Indo-European | 96 | 0.01 | Present study |
| 26 | Warli | West | Maharashtra | Indo-European | 100 | 0.26 | Present study |
| 27 | Mupanar | South | Tamil Nadu | Dravidian | 99 | 0.394 | Present study |
| 28 | Saxena | Central | Madhya Pradesh | Indo-European | 96 | 0.135 | Present study |
| 29 | Jain | Northwest | Rajasthan | Indo-European | 92 | 0.196 | Present study |
| 30 | Paliyar | South | Tamil Nadu | Dravidian | 96 | 0.146 | Present study |
| 31 | Gamit | Northwest | Gujarat | Indo-European | 74 | 0.23 | Present study |
| 32 | Arunthathier | South | Tamil Nadu | Dravidian | 85 | 0.212 | Present study |
| 33 | Lohar | South | Andhra Pradesh | Dravidian | 48 | 0.313 | Present study |
| 34 | Padmashali | South | Andhra Pradesh | Dravidian | 33 | 0.152 | Present study |
| 35 | Kuruva | South | Andhra Pradesh | Dravidian | 33 | 0.303 | Present study |
| 36 | Siddi Karnataka | South | Karnataka | Dravidian | 44 | 0.205 | Present study |
| 37 | Mahadeo Koli | West | Maharashtra | Indo-European | 50 | 0.18 | Present study |
| 38 | Thakur | West | Maharashtra | Indo-European | 48 | 0.083 | Present study |
| 39 | Bhil | Central | Madhya Pradesh | Indo-European | 30 | 0.5 | Present study |
| 40 | Bharia | Central | Madhya Pradesh | Dravidian | 50 | 0.1 | Present study |
| 41 | Sahariya | Central | Madhya Pradesh | Indo-European | 73 | 0.014 | Present study |
| 42 | Pal | North | Uttar Pradesh | Indo-European | 47 | 0.17 | Present study |
| 43 | Shia | North | Uttar Pradesh | Muslim Religious Group | 48 | 0.083 | Present study |
| 44 | Syed | South | Andhra Pradesh | Muslim Religious Group | 44 | 0.136 | Present study |
| 45 | Vadde | South | Andhra Pradesh | Dravidian | 29 | 0.138 | Present study |
| 46 | Ao Naga | Northeast | Nagaland | Tibeto-Burman | 60 | 0 | Present study |
| 47 | Cheksang Naga | Northeast | Nagaland | Tibeto-Burman | 55 | 0 | Present study |
| 48 | Banraut | North | Uttarakhand | Tibeto-Burman | 38 | 0 | Present study |
| 49 | Poumai Naga | Northeast | Manipur | Tibeto-Burman | 50 | 0 | Present study |
| 50 | Naga Sema | Northeast | Nagaland | Tibeto-Burman | 54 | 0 | Present study |
| 51 | Changpa | North | Jammu & Kashmir | Tibeto-Burman | 50 | 0 | Present study |
| 52 | Mizo | Northeast | Mizoram | Tibeto-Burman | 46 | 0 | Present study |
| 53 | Siddi Gujarat | Northwest | Gujarat | Indo-European | 60 | 0 | Present study |
| 54 | Khasi | Northeast | Meghalaya | Austroasiatic | 40 | 0 | Present study |
| 55 | Chakma | Northeast | Bangladesh | Tibeto-Burman | 86 | 0 | Present study |
| 56 | Marma | Northeast | Bangladesh | Tibeto-Burman | 59 | 0 | Present study |
| 57 | Tripura | Northeast | Bangladesh | Tibeto-Burman | 84 | 0 | Present study |
| 58 | Onge | Andaman | Andaman | Andamanese | 10 | 0 | Thangaraj et al. 2003 |
| 59 | Great Andamanese | Andaman | Andaman | Andamanese | 10 | 0 | Thangaraj et al. 2003 |
| 63 | Andhra Brahmin | South | Andhra Pradesh | Dravidian | 19 | 0.158 | Trivedi et al. 2008 |
| 64 | Chenchu | South | Andhra Pradesh | Dravidian | 20 | 0 | Trivedi et al. 2008 |
| 65 | Kamma Chaudhary | South | Andhra Pradesh | Dravidian | 19 | 0.053 | Trivedi et al. 2008 |
| 66 | Kapu Naidu | South | Andhra Pradesh | Dravidian | 20 | 0 | Trivedi et al. 2008 |
| 67 | Komati | South | Andhra Pradesh | Dravidian | 20 | 0 | Trivedi et al. 2008 |
| 68 | Lambadi | South | Andhra Pradesh | Dravidian | 20 | 0.05 | Trivedi et al. 2008 |
| 69 | Naikpod Gond | South | Andhra Pradesh | Dravidian | 20 | 0.4 | Trivedi et al. 2008 |
| 70 | Raju | South | Andhra Pradesh | Dravidian | 19 | 0 | Trivedi et al. 2008 |
| 71 | Reddy | South | Andhra Pradesh | Dravidian | 14 | 0 | Trivedi et al. 2008 |
| 72 | Yerukula | South | Andhra Pradesh | Dravidian | 20 | 0 | Trivedi et al. 2008 |
| 73 | Adi Pasi | Northeast | Arunachal Pradesh | Tibeto-Burman | 10 | 0 | Trivedi et al. 2008 |
| 74 | Bihar Brahmin | East | Bihar | Indo-European | 18 | 0 | Trivedi et al. 2008 |
| 75 | Bhumihar | East | Bihar | Indo-European | 20 | 0.1 | Trivedi et al. 2008 |
| 76 | Rajput | East | Bihar | Indo-European | 12 | 0.083 | Trivedi et al. 2008 |
| 77 | Kayastha | East | Bihar | Indo-European | 14 | 0.286 | Trivedi et al. 2008 |
| 78 | Yadava | East | Bihar | Indo-European | 8 | 0.125 | Trivedi et al. 2008 |
| 79 | Kurmi | East | Bihar | Indo-European | 13 | 0.308 | Trivedi et al. 2008 |
| 80 | Baniya | East | Bihar | Indo-European | 11 | 0.364 | Trivedi et al. 2008 |
| 81 | Patel | Northwest | Gujarat | Indo-European | 9 | 0.444 | Trivedi et al. 2008 |
| 82 | Rajput | North | Himanchal Pradesh | Indo-European | 15 | 0.133 | Trivedi et al. 2008 |
| 83 | Ho | East | Jharkhand | Austroasiatic | 12 | 0 | Trivedi et al. 2008 |
| 84 | Bhumij | East | Jharkhand | Austroasiatic | 15 | 0 | Trivedi et al. 2008 |
| 85 | Kharia | East | Jharkhand | Austroasiatic | 10 | 0 | Trivedi et al. 2008 |
| 86 | Munda | East | Jharkhand | Austroasiatic | 14 | 0 | Trivedi et al. 2008 |
| 87 | Birhor | East | Jharkhand | Austroasiatic | 15 | 0 | Trivedi et al. 2008 |
| 88 | Santhal | East | Jharkhand | Austroasiatic | 15 | 0 | Trivedi et al. 2008 |
| 89 | Iyenger | South | Karnataka | Dravidian | 19 | 0.421 | Trivedi et al. 2008 |
| 90 | Lingayat | South | Karnataka | Dravidian | 12 | 0.333 | Trivedi et al. 2008 |
| 91 | Gowda | South | Karnataka | Dravidian | 7 | 0.143 | Trivedi et al. 2008 |
| 92 | Bhovi | South | Karnataka | Dravidian | 15 | 0.467 | Trivedi et al. 2008 |
| 93 | Christian | South | Karnataka | Dravidian | 13 | 0.231 | Trivedi et al. 2008 |
| 94 | Muslim | South | Karnataka | Dravidian | 4 | 0.25 | Trivedi et al. 2008 |
| 95 | Kuruva | South | Karnataka | Dravidian | 13 | 0.231 | Trivedi et al. 2008 |
| 96 | Desath Brahmin | West | Maharashtra | Indo-European | 19 | 0.158 | Trivedi et al. 2008 |
| 97 | Chitpavana Brahmin | West | Maharashtra | Indo-European | 15 | 0.2 | Trivedi et al. 2008 |
| 98 | Maratha | West | Maharashtra | Indo-European | 16 | 0.375 | Trivedi et al. 2008 |
| 99 | Dhangar | West | Maharashtra | Indo-European | 16 | 0.313 | Trivedi et al. 2008 |
| 100 | Pawara | West | Maharashtra | Indo-European | 16 | 0.5 | Trivedi et al. 2008 |
| 101 | Katkari | West | Maharashtra | Indo-European | 19 | 0.368 | Trivedi et al. 2008 |
| 102 | Madia Gond | West | Maharashtra | Dravidian | 14 | 0.571 | Trivedi et al. 2008 |
| 103 | Mahadeo Koli | West | Maharashtra | Indo-European | 11 | 0.364 | Trivedi et al. 2008 |
| 104 | Mara | Northeast | Mizoram | Tibeto-Burman | 15 | 0 | Trivedi et al. 2008 |
| 105 | Hmar | Northeast | Mizoram | Tibeto-Burman | 20 | 0 | Trivedi et al. 2008 |
| 106 | Lai | Northeast | Mizoram | Tibeto-Burman | 12 | 0 | Trivedi et al. 2008 |
| 107 | Lusei | Northeast | Mizoram | Tibeto-Burman | 19 | 0 | Trivedi et al. 2008 |
| 108 | Kuki | Northeast | Mizoram | Tibeto-Burman | 12 | 0 | Trivedi et al. 2008 |
| 109 | Manipuri Muslim | Northeast | Mizoram | Tibeto-Burman | 9 | 0 | Trivedi et al. 2008 |
| 110 | Oriya Brahmin | East | Orissa | Indo-European | 24 | 0.083 | Trivedi et al. 2008 |
| 111 | Karan | East | Orissa | Indo-European | 18 | 0.167 | Trivedi et al. 2008 |
| 112 | Khandayat | East | Orissa | Indo-European | 13 | 0.231 | Trivedi et al. 2008 |
| 113 | Gope | East | Orissa | Indo-European | 16 | 0.125 | Trivedi et al. 2008 |
| 114 | Paroja | East | Orissa | Dravidian | 15 | 0.2 | Trivedi et al. 2008 |
| 115 | Juang | East | Orissa | Austroasiatic | 20 | 0 | Trivedi et al. 2008 |
| 116 | Saora | East | Orissa | Austroasiatic | 19 | 0.211 | Trivedi et al. 2008 |
| 117 | Chakkliar | South | Tamil Nadu | Dravidian | 14 | 0.286 | Trivedi et al. 2008 |
| 118 | Kallar | South | Tamil Nadu | Dravidian | 12 | 0.167 | Trivedi et al. 2008 |
| 119 | Vanniyar | South | Tamil Nadu | Dravidian | 13 | 0.308 | Trivedi et al. 2008 |
| 120 | Pallar | South | Tamil Nadu | Dravidian | 16 | 0.5 | Trivedi et al. 2008 |
| 121 | Gounder | South | Tamil Nadu | Dravidian | 18 | 0.444 | Trivedi et al. 2008 |
| 122 | Irular | South | Tamil Nadu | Dravidian | 12 | 0.5 | Trivedi et al. 2008 |
| 123 | Kanyakubja Brahmin | North | Uttar Pradesh | Indo-European | 11 | 0.182 | Trivedi et al. 2008 |
| 124 | Jat | North | Uttar Pradesh | Indo-European | 10 | 0 | Trivedi et al. 2008 |
| 125 | Thakur | North | Uttar Pradesh | Indo-European | 8 | 0.375 | Trivedi et al. 2008 |
| 126 | Khatri | North | Uttar Pradesh | Indo-European | 7 | 0 | Trivedi et al. 2008 |
| 127 | Bhoska | North | Uttar Pradesh | Indo-European | 10 | 0.2 | Trivedi et al. 2008 |
| 128 | Kurmi | North | Uttar Pradesh | Indo-European | 11 | 0 | Trivedi et al. 2008 |
| 129 | Jaunsari | North | Uttar Pradesh | Indo-European | 6 | 0 | Trivedi et al. 2008 |
| 130 | Mahisiya | East | West Bengal | Indo-European | 20 | 0.1 | Trivedi et al. 2008 |
| 131 | Namasudra | East | West Bengal | Indo-European | 20 | 0.15 | Trivedi et al. 2008 |
| 132 | Bauri | East | West Bengal | Indo-European | 20 | 0 | Trivedi et al. 2008 |
| 133 | Maheli | East | West Bengal | Austroasiatic | 20 | 0.35 | Trivedi et al. 2008 |
| 134 | Karmali | East | West Bengal | Austroasiatic | 19 | 0 | Trivedi et al. 2008 |
| 135 | Lodha | East | West Bengal | Austroasiatic | 20 | 0.1 | Trivedi et al. 2008 |
| 136 | Ezhava Hindu | South | Kerala | Dravidian | 9 | 0 | Trivedi et al. 2008 |
| 137 | Nair | South | Kerala | Dravidian | 4 | 0 | Trivedi et al. 2008 |
| 138 | Bengali | East | West Bengal | Indo-European | 30 | 0.1 | Kivisild et al. 2003 |
| 139 | Chenchu | South | Andhra Pradesh | Dravidian | 41 | 0.366 | Kivisild et al. 2003 |
| 140 | Cobra Br | West | Maharashtra | Indo-European | 44 | 0.068 | Kivisild et al. 2003 |
| 141 | Gujarat | Northwest | Gujarat | Indo-European | 29 | 0.103 | Kivisild et al. 2003 |
| 142 | Koya | South | Andhra Pradesh | Dravidian | 42 | 0.595 | Kivisild et al. 2003 |
| 143 | Lambadi | South | Andhra Pradesh | Dravidian | 38 | 0.079 | Kivisild et al. 2003 |
| 144 | Punjab | Northwest | Punjab | Indo-European | 66 | 0.03 | Kivisild et al. 2003 |
| 145 | Khasi | Northeast | Meghalaya | Mon-Khmer | 354 | 0 | Reddy et al. 2007 |
| 146 | Khasi | Northeast | Meghalaya | Mon-Khmer | 21 | 0 | Chaubey et al. 2011 |
| 147 | Bhar | North | Uttar Pradesh | Indo-European | 22 | 0.545 | Chaubey et al. 2008 |
| 148 | Cheek Baraik | East | Jharkhand | Indo-European | 20 | 0 | Chaubey et al. 2008 |
| 149 | Dusadh | East | Bihar | Indo-European | 22 | 0 | Chaubey et al. 2008 |
| 150 | Harijan | North | Uttar Pradesh | Indo-European | 20 | 0 | Chaubey et al. 2008 |
| 151 | Ho | East | Bihar | Austroasiatic | 25 | 0.24 | Chaubey et al. 2008 |
| 152 | Kanwar | Central | Madhya Pradesh | Indo-European | 19 | 0.632 | Chaubey et al. 2008 |
| 153 | Kharia | Central | Madhya Pradesh | Austroasiatic | 21 | 0.048 | Chaubey et al. 2008 |
| 154 | Lohra | East | Bihar | Indo-European | 16 | 0.688 | Chaubey et al. 2008 |
| 155 | Mawasi | Central | Madhya Pradesh | Austroasiatic | 24 | 0.083 | Chaubey et al. 2008 |
| 156 | Munda | East | Bihar | Austroasiatic | 42 | 0.31 | Chaubey et al. 2008 |
| 157 | Santhal | Central | Madhya Pradesh | Austroasiatic | 21 | 0.048 | Chaubey et al. 2008 |
| 158 | Asur | East | Jharkhand | Austroasiatic | 88 | 0 | Chaubey et al. 2011 |
| 159 | Ho | East | Bihar | Austroasiatic | 45 | 0 | Chaubey et al. 2011 |
| 160 | Mawasi_JHK | East | Jharkhand | Austroasiatic | 27 | 0 | Chaubey et al. 2011 |
| 161 | Mawasi_MP | Central | Madhya Pradesh | Austroasiatic | 12 | 0 | Chaubey et al. 2011 |
| 162 | Mahali | East | Jharkhand | Austroasiatic | 32 | 0 | Chaubey et al. 2011 |
| 163 | Santhal | East | Jharkhand | Austroasiatic | 20 | 0 | Chaubey et al. 2011 |
| 164 | Birhor_CG | East | Chhattisgarh | Austroasiatic | 27 | 0 | Chaubey et al. 2011 |
| 165 | Birhor_MAH | West | Maharashtra | Austroasiatic | 35 | 0 | Chaubey et al. 2011 |
| 166 | Baiga_MP | Central | Madhya Pradesh | Austroasiatic | 23 | 0 | Chaubey et al. 2011 |
| 167 | Baiga_OR | East | Orissa | Austroasiatic | 42 | 0 | Chaubey et al. 2011 |
| 168 | Bonda | East | Orissa | Austroasiatic | 42 | 0 | Chaubey et al. 2011 |
| 169 | Gadaba | East | Orissa | Austroasiatic | 27 | 0 | Chaubey et al. 2011 |
| 170 | Juang | East | Orissa | Austroasiatic | 54 | 0 | Chaubey et al. 2011 |
| 171 | Kharia | East | Chhattisgarh | Austroasiatic | 37 | 0 | Chaubey et al. 2011 |
| 172 | Savara | East | Orissa | Austroasiatic | 21 | 0 | Chaubey et al. 2011 |
| 173 | Garo | Northeast | Meghalaya | Tibeto-Burman | 25 | 0 | Chaubey et al. 2011 |
| 174 | Oraon | East | Jharkhand | Dravidian | 12 | 0 | Trivedi et al. 2008 |
| 175 | Satnami | Central | Madhya Pradesh | Indo-European | 18 | 0.222 | Chaubey et al. 2008 |
| 176 | Kora | East | West Bengal | Indo-European | 20 | 0.35 | Trivedi et al. 2008 |
| 177 | Moor | South | Sri Lanka | Indo-European | 44 | 0.159 | Kivisild et al. 2003 |
| 178 | Singali | South | Sri Lanka | Indo-European | 34 | 0.088 | Kivisild et al. 2003 |
| 179 | Mahadeo Koli | West | Maharashtra | Indo-European | 50 | 0 | Thangaraj et al. 2010 |
| 180 | Thakar | West | Maharashtra | Indo-European | 48 | 0 | Thangaraj et al. 2010 |
| 181 | Brahmin | Central | Indian mixed | Indo-European | 17 | 0.176 | Zerjal et al. 2007 |
| 182 | Kshathriya | Central | Indian mixed | Indo-European | 19 | 0.211 | Zerjal et al. 2007 |
| 183 | Vaishya | Central | Indian mixed | Indo-European | 8 | 0 | Zerjal et al. 2007 |
| 184 | Sudra | Central | Indian mixed | Indo-European | 23 | 0.174 | Zerjal et al. 2007 |
| 185 | Panchama | Central | Indian mixed | Indo-European | 19 | 0.158 | Zerjal et al. 2007 |
| 186 | Brahmin_J | North | Uttar Pradesh | Indo-European | 20 | 0.65 | Zerjal et al. 2007 |
| 187 | Kshathriya_J | North | Uttar Pradesh | Indo-European | 47 | 0.043 | Zerjal et al. 2007 |
| 188 | Vaishya_J | North | Uttar Pradesh | Indo-European | 39 | 0.205 | Zerjal et al. 2007 |
| 189 | Sudra_J | North | Uttar Pradesh | Indo-European | 7 | 0 | Zerjal et al. 2007 |
| 190 | Panchama_J | North | Uttar Pradesh | Indo-European | 28 | 0.214 | Zerjal et al. 2007 |
| 191 | Charan | Northwest | Gujarat | Indo-European | 42 | 0 | Shah et al. 2011 |
| 192 | Bharwad | Northwest | Gujarat | Indo-European | 48 | 0.333 | Shah et al. 2011 |
| 193 | Medar | South | Karnataka | Dravidian | 56 | 0.411 | Shah et al. 2011 |
| 194 | Gram Vokkal | South | Karnataka | Dravidian | 54 | 0.296 | Shah et al. 2011 |
| 195 | Korava | South | Karnataka | Dravidian | 38 | 0 | Shah et al. 2011 |
| 196 | Kare Vokkal | South | Karnataka | Dravidian | 30 | 0 | Shah et al. 2011 |
| 197 | Gujarat | Northwest | Gujarat | Indo-European | 36 | 0.194 | Kivisild et al. 2003 |
| 198 | Koraga | Northwest | Gujarat | Indo-European | 31 | 0.935 | Kivisild et al. 2003 |
| 199 | Bharia | Central | Madhya Pradesh | Dravidian | 50 | 0.1 | Sharma et al. 2012 |
| 200 | Bhil | Central | Madhya Pradesh | Indo-European | 30 | 0.5 | Sharma et al. 2012 |
| 201 | Saharia | Central | Madhya Pradesh | Indo-European | 73 | 0.014 | Sharma et al. 2012 |
| 202 | Kol | East | West Bengal | Austroasiatic | 62 | 0.113 | Debnath et al. 2012 |
| 203 | Santhal | East | West Bengal | Austroasiatic | 51 | 0.176 | Debnath et al. 2012 |
| 204 | Kharia | East | West Bengal | Austroasiatic | 34 | 0.059 | Debnath et al. 2012 |
| 205 | Dhimal | East | West Bengal | Tibeto-Burman | 36 | 0.083 | Debnath et al. 2012 |
| 206 | Rabha | East | West Bengal | Tibeto-Burman | 26 | 0.038 | Debnath et al. 2012 |
| 207 | Mech | East | West Bengal | Tibeto-Burman | 19 | 0 | Debnath et al. 2012 |
| 208 | Lachungpa | Northeast | Sikkim | Tibeto-Burman | 11 | 0.091 | Debnath et al. 2012 |
| 209 | Oraon | East | West Bengal | Dravidian | 31 | 0.226 | Debnath et al. 2012 |
| 210 | Bengali | East | West Bengal | Indo-European | 54 | 0.13 | Debnath et al. 2012 |
| 211 | Rajbanshi | East | West Bengal | Indo-European | 51 | 0.02 | Debnath et al. 2012 |
| 212 | Tharu-C I | Nepal | Nepal | Indo-European | 57 | 0.07 | Fornarino et al. 2009 |
| 213 | Tharu-C II | Nepal | Nepal | Indo-European | 77 | 0.182 | Fornarino et al. 2009 |
| 214 | Tharu-E | Nepal | Nepal | Indo-European | 37 | 0.054 | Fornarino et al. 2009 |
| 215 | Hindu-Terai | Nepal | Nepal | Indo-European | 26 | 0.038 | Fornarino et al. 2009 |
| 216 | Hindu-N Delhi | North | New Delhi | Indo-European | 49 | 0.102 | Fornarino et al. 2009 |
| 217 | Tribe AP | South | Andhra Pradesh | Dravidian | 29 | 0.276 | Fornarino et al. 2009 |
