# Supplementary material for: The Phylogeography of Y-Chromosome Haplogroup H1a1a-M82 Reveals the Likely Indian Origin of the European Romani Populations
Source: PLoS One. 2012 Nov 28;7(11):e48477. doi: 10.1371/journal.pone.0048477 (PMC3509117; doi:10.1371/journal.pone.0048477)
Supplement: Table S2 — The 15 loci Y-STR profile of haplogroup H1a1a-M82 belonging to Indian, Afghani and European Roma population, used in the present analysis. (DOC) [file pone.0048477.s004.doc]

| **Grouping** | **State** | **Language** | **Id** | **DYS19** | **DYS389ab** | **DYS389cd** | **DYS390** | **DYS391** | **DYS392** | **DYS393** | **DYS437** | **DYS438** | **DYS439** | **DYS448** | **DYS456** | **DYS458** | **DYS635** | **H4** | **References** |
| --- | --- | --- | --- | --- | --- | --- | --- | --- | --- | --- | --- | --- | --- | --- | --- | --- | --- | --- | --- |
| Afghani | Afghanistan | Indo-European | Afg19 | 15 | 16 | 13 | 24 | 10 | 11 | 12 | 14 | 9 | 11 | 18 | 15 | 16 | 21 | 11 | Lacau et al. 2012 |
| Afghani | Afghanistan | Indo-European | Afg22 | 15 | 16 | 13 | 24 | 10 | 11 | 12 | 14 | 9 | 11 | 18 | 15 | 16 | 21 | 11 | Lacau et al. 2012 |
| Afghani | Afghanistan | Indo-European | Afg25 | 15 | 16 | 13 | 24 | 10 | 11 | 12 | 14 | 9 | 11 | 18 | 15 | 16 | 22 | 11 | Lacau et al. 2012 |
| Afghani | Afghanistan | Indo-European | Afg87 | 14 | 16 | 12 | 22 | 10 | 11 | 12 | 14 | 10 | 12 | 19 | 16 | 17 | 17 | 11 | Lacau et al. 2012 |
| Afghani | Afghanistan | Indo-European | Afg126 | 15 | 16 | 13 | 24 | 10 | 11 | 12 | 14 | 9 | 11 | 18 | 15 | 16 | 21 | 11 | Lacau et al. 2012 |
| Afghani | Afghanistan | Indo-European | Afg171 | 14 | 17 | 13 | 22 | 10 | 11 | 12 | 14 | 10 | 10 | 19 | 15 | 17 | 17 | 12 | Lacau et al. 2012 |
| Europe | Croatia | Indo-European (Romani) | Bar1 | 14 | 16 | 14 | 22 | 10 | 11 | 12 | 14 | 9 | 11 | 19 | 15 | 18 | 20 | 12 | Klaric et al. 2009 |
| Europe | Croatia | Indo-European (Romani) | Bar2 | 15 | 16 | 14 | 22 | 10 | 11 | 12 | 14 | 9 | 11 | 18 | 15 | 17 | 20 | 12 | Klaric et al. 2009 |
| Europe | Croatia | Indo-European (Romani) | Bar3 | 15 | 16 | 14 | 22 | 10 | 11 | 12 | 14 | 9 | 11 | 18 | 15 | 17 | 20 | 12 | Klaric et al. 2009 |
| Europe | Croatia | Indo-European (Romani) | Bar4 | 15 | 16 | 14 | 22 | 10 | 11 | 12 | 14 | 9 | 11 | 18 | 15 | 17 | 20 | 12 | Klaric et al. 2009 |
| Europe | Croatia | Indo-European (Romani) | Bar5 | 15 | 16 | 14 | 22 | 10 | 11 | 12 | 14 | 9 | 11 | 18 | 15 | 17 | 20 | 12 | Klaric et al. 2009 |
| Europe | Croatia | Indo-European (Romani) | Bar6 | 15 | 16 | 14 | 22 | 10 | 11 | 12 | 14 | 9 | 11 | 18 | 15 | 17 | 20 | 12 | Klaric et al. 2009 |
| Europe | Croatia | Indo-European (Romani) | Bar7 | 15 | 16 | 14 | 22 | 10 | 11 | 12 | 14 | 9 | 11 | 18 | 15 | 17 | 20 | 12 | Klaric et al. 2009 |
| Europe | Croatia | Indo-European (Romani) | Bar8 | 15 | 16 | 14 | 22 | 10 | 11 | 12 | 14 | 9 | 11 | 18 | 15 | 17 | 20 | 12 | Klaric et al. 2009 |
| Europe | Croatia | Indo-European (Romani) | Bar9 | 15 | 16 | 13 | 22 | 10 | 11 | 12 | 14 | 9 | 11 | 19 | 15 | 17 | 20 | 12 | Klaric et al. 2009 |
| Europe | Croatia | Indo-European (Romani) | Bar10 | 15 | 16 | 13 | 22 | 10 | 11 | 12 | 14 | 9 | 11 | 19 | 15 | 17 | 20 | 12 | Klaric et al. 2009 |
| Europe | Croatia | Indo-European (Romani) | Bar11 | 15 | 16 | 14 | 22 | 10 | 11 | 12 | 14 | 9 | 11 | 18 | 15 | 16 | 20 | 12 | Klaric et al. 2009 |
| Europe | Croatia | Indo-European (Romani) | Bar12 | 15 | 16 | 14 | 22 | 10 | 11 | 12 | 14 | 9 | 11 | 18 | 15 | 16 | 20 | 12 | Klaric et al. 2009 |
| Europe | Croatia | Indo-European (Romani) | Bar13 | 15 | 16 | 14 | 22 | 10 | 11 | 12 | 14 | 9 | 11 | 18 | 15 | 16 | 20 | 12 | Klaric et al. 2009 |
| Europe | Croatia | Indo-European (Romani) | Bar14 | 15 | 16 | 14 | 22 | 10 | 11 | 12 | 14 | 9 | 11 | 18 | 15 | 16 | 20 | 12 | Klaric et al. 2009 |
| Europe | Croatia | Indo-European (Romani) | Bar15 | 15 | 16 | 14 | 22 | 10 | 11 | 12 | 14 | 9 | 12 | 18 | 16 | 16 | 20 | 12 | Klaric et al. 2009 |
| Europe | Croatia | Indo-European (Romani) | Bar16 | 15 | 16 | 14 | 22 | 11 | 11 | 12 | 14 | 9 | 11 | 18 | 15 | 17 | 20 | 12 | Klaric et al. 2009 |
| Europe | Croatia | Indo-European (Romani) | Bar17 | 15 | 17 | 14 | 22 | 9 | 11 | 12 | 14 | 9 | 11 | 18 | 15 | 17 | 20 | 12 | Klaric et al. 2009 |
| Europe | Croatia | Indo-European (Romani) | Bar18 | 15 | 16 | 15 | 22 | 10 | 11 | 12 | 14 | 9 | 11 | 18 | 15 | 17 | 20 | 12 | Klaric et al. 2009 |
| Europe | Croatia | Indo-European (Romani) | Bar19 | 15 | 17 | 14 | 22 | 9 | 11 | 12 | 14 | 9 | 11 | 18 | 15 | 17 | 20 | 12 | Klaric et al. 2009 |
| Europe | Croatia | Indo-European (Romani) | Bar20 | 15 | 16 | 14 | 22 | 10 | 11 | 12 | 14 | 9 | 11 | 19 | 15 | 17 | 20 | 12 | Klaric et al. 2009 |
| Europe | Croatia | Indo-European (Romani) | Bar21 | 15 | 16 | 12 | 23 | 10 | 12 | 12 | 14 | 9 | 12 | 19 | 14 | 15 | 21 | 12 | Klaric et al. 2009 |
| Europe | Croatia | Indo-European (Romani) | Bar22 | 15 | 16 | 14 | 22 | 10 | 11 | 12 | 14 | 9 | 11 | 19 | 15 | 18 | 20 | 12 | Klaric et al. 2009 |
| Europe | Croatia | Indo-European (Romani) | Bar23 | 15 | 16 | 14 | 22 | 10 | 11 | 12 | 14 | 9 | 11 | 19 | 15 | 18 | 20 | 12 | Klaric et al. 2009 |
| Europe | Croatia | Indo-European (Romani) | Bar24 | 15 | 16 | 14 | 22 | 10 | 11 | 12 | 14 | 9 | 11 | 18 | 15 | 17 | 20 | 12 | Klaric et al. 2009 |
| Europe | Croatia | Indo-European (Romani) | Bar25 | 15 | 16 | 14 | 22 | 10 | 11 | 12 | 14 | 9 | 11 | 18 | 15 | 17 | 20 | 12 | Klaric et al. 2009 |
| Europe | Croatia | Indo-European (Romani) | Bar26 | 15 | 16 | 14 | 22 | 10 | 11 | 12 | 14 | 9 | 11 | 18 | 15 | 17 | 20 | 12 | Klaric et al. 2009 |
| Europe | Croatia | Indo-European (Romani) | Bar27 | 15 | 16 | 14 | 22 | 10 | 11 | 12 | 14 | 9 | 11 | 18 | 15 | 17 | 20 | 12 | Klaric et al. 2009 |
| Europe | Croatia | Indo-European (Romani) | Bar28 | 15 | 16 | 14 | 22 | 10 | 11 | 12 | 14 | 9 | 11 | 18 | 15 | 17 | 20 | 12 | Klaric et al. 2009 |
| Europe | Croatia | Indo-European (Romani) | Bar29 | 15 | 16 | 14 | 22 | 10 | 11 | 12 | 14 | 9 | 11 | 18 | 15 | 17 | 20 | 12 | Klaric et al. 2009 |
| Europe | Croatia | Indo-European (Romani) | Bar30 | 15 | 16 | 14 | 22 | 10 | 11 | 12 | 14 | 9 | 11 | 18 | 15 | 17 | 20 | 12 | Klaric et al. 2009 |
| Europe | Croatia | Indo-European (Romani) | Bar31 | 15 | 16 | 14 | 22 | 10 | 11 | 12 | 14 | 9 | 11 | 18 | 15 | 17 | 20 | 12 | Klaric et al. 2009 |
| Europe | Croatia | Indo-European (Romani) | Bar32 | 15 | 16 | 14 | 22 | 10 | 11 | 12 | 14 | 9 | 11 | 18 | 15 | 17 | 20 | 12 | Klaric et al. 2009 |
| Europe | Croatia | Indo-European (Romani) | Bar33 | 15 | 16 | 14 | 22 | 10 | 11 | 12 | 14 | 9 | 11 | 18 | 15 | 17 | 20 | 12 | Klaric et al. 2009 |
| Europe | Croatia | Indo-European (Romani) | Bar34 | 15 | 16 | 14 | 22 | 10 | 11 | 12 | 14 | 9 | 11 | 18 | 15 | 17 | 20 | 12 | Klaric et al. 2009 |
| Europe | Croatia | Indo-European (Romani) | Bar35 | 15 | 16 | 14 | 22 | 10 | 11 | 12 | 14 | 9 | 11 | 18 | 15 | 17 | 20 | 12 | Klaric et al. 2009 |
| Europe | Croatia | Indo-European (Romani) | Bar36 | 15 | 16 | 14 | 22 | 10 | 11 | 12 | 14 | 9 | 11 | 18 | 15 | 17 | 20 | 12 | Klaric et al. 2009 |
| Europe | Croatia | Indo-European (Romani) | Bar37 | 15 | 16 | 14 | 22 | 10 | 11 | 12 | 14 | 9 | 11 | 18 | 15 | 17 | 20 | 12 | Klaric et al. 2009 |
| Europe | Croatia | Indo-European (Romani) | Bar38 | 15 | 16 | 14 | 22 | 10 | 11 | 12 | 14 | 9 | 11 | 18 | 15 | 17 | 20 | 12 | Klaric et al. 2009 |
| Europe | Croatia | Indo-European (Romani) | Bar39 | 15 | 16 | 14 | 22 | 10 | 11 | 12 | 14 | 9 | 11 | 18 | 15 | 17 | 20 | 12 | Klaric et al. 2009 |
| Europe | Croatia | Indo-European (Romani) | Bar40 | 15 | 16 | 14 | 22 | 10 | 11 | 12 | 14 | 9 | 11 | 18 | 15 | 17 | 20 | 12 | Klaric et al. 2009 |
| Europe | Croatia | Indo-European (Romani) | Bar41 | 15 | 16 | 14 | 22 | 10 | 11 | 12 | 14 | 9 | 11 | 18 | 15 | 17 | 20 | 12 | Klaric et al. 2009 |
| Europe | Croatia | Indo-European (Romani) | Bar42 | 15 | 16 | 14 | 22 | 10 | 11 | 12 | 14 | 9 | 11 | 18 | 15 | 17 | 20 | 12 | Klaric et al. 2009 |
| Europe | Croatia | Indo-European (Romani) | Bar43 | 15 | 16 | 14 | 22 | 10 | 11 | 12 | 14 | 9 | 11 | 18 | 15 | 17 | 20 | 12 | Klaric et al. 2009 |
| Europe | Croatia | Indo-European (Romani) | Bar44 | 15 | 16 | 14 | 22 | 10 | 11 | 12 | 14 | 9 | 11 | 18 | 15 | 17 | 20 | 12 | Klaric et al. 2009 |
| Europe | Croatia | Indo-European (Romani) | Bar45 | 15 | 16 | 14 | 22 | 10 | 11 | 12 | 14 | 9 | 11 | 18 | 15 | 17 | 20 | 12 | Klaric et al. 2009 |
| Europe | Croatia | Indo-European (Romani) | Bar46 | 15 | 16 | 14 | 22 | 10 | 11 | 12 | 14 | 9 | 11 | 18 | 15 | 17 | 20 | 12 | Klaric et al. 2009 |
| Europe | Croatia | Indo-European (Romani) | Bar47 | 15 | 16 | 14 | 22 | 10 | 11 | 12 | 14 | 9 | 11 | 18 | 15 | 17 | 20 | 12 | Klaric et al. 2009 |
| Europe | Croatia | Indo-European (Romani) | Bar48 | 15 | 16 | 14 | 22 | 10 | 11 | 12 | 14 | 9 | 11 | 18 | 15 | 17 | 20 | 12 | Klaric et al. 2009 |
| Europe | Croatia | Indo-European (Romani) | Med1 | 15 | 16 | 15 | 22 | 10 | 11 | 12 | 14 | 9 | 11 | 19 | 15 | 18 | 20 | 12 | Klaric et al. 2009 |
| Europe | Croatia | Indo-European (Romani) | Med2 | 16 | 16 | 14 | 22 | 10 | 11 | 12 | 14 | 9 | 11 | 18 | 15 | 17 | 20 | 12 | Klaric et al. 2009 |
| Europe | Croatia | Indo-European (Romani) | Med3 | 16 | 16 | 14 | 22 | 10 | 11 | 12 | 14 | 9 | 11 | 18 | 15 | 17 | 20 | 12 | Klaric et al. 2009 |
| Europe | Croatia | Indo-European (Romani) | Med4 | 16 | 16 | 14 | 22 | 10 | 11 | 12 | 14 | 9 | 11 | 18 | 15 | 17 | 20 | 12 | Klaric et al. 2009 |
| Europe | Croatia | Indo-European (Romani) | Med5 | 15 | 16 | 15 | 22 | 10 | 11 | 12 | 14 | 9 | 11 | 19 | 15 | 18 | 20 | 12 | Klaric et al. 2009 |
| Europe | Croatia | Indo-European (Romani) | Med6 | 15 | 16 | 15 | 22 | 10 | 11 | 12 | 14 | 9 | 11 | 19 | 15 | 18 | 20 | 12 | Klaric et al. 2009 |
| Europe | Croatia | Indo-European (Romani) | Med7 | 15 | 16 | 15 | 22 | 10 | 11 | 12 | 14 | 9 | 11 | 19 | 15 | 18 | 20 | 12 | Klaric et al. 2009 |
| Europe | Croatia | Indo-European (Romani) | Med8 | 15 | 16 | 14 | 22 | 10 | 11 | 12 | 14 | 9 | 11 | 19 | 15 | 18 | 20 | 12 | Klaric et al. 2009 |
| Europe | Croatia | Indo-European (Romani) | Med9 | 15 | 16 | 14 | 22 | 10 | 11 | 12 | 14 | 9 | 11 | 19 | 15 | 18 | 20 | 12 | Klaric et al. 2009 |
| Europe | Croatia | Indo-European (Romani) | Med10 | 15 | 16 | 14 | 22 | 10 | 11 | 12 | 14 | 9 | 11 | 19 | 15 | 18 | 20 | 12 | Klaric et al. 2009 |
| Europe | Croatia | Indo-European (Romani) | Med11 | 15 | 16 | 14 | 22 | 10 | 11 | 12 | 14 | 9 | 11 | 18 | 15 | 17 | 20 | 12 | Klaric et al. 2009 |
| Europe | Croatia | Indo-European (Romani) | Med12 | 15 | 16 | 14 | 22 | 10 | 11 | 12 | 14 | 9 | 11 | 18 | 15 | 17 | 20 | 12 | Klaric et al. 2009 |
| Europe | Croatia | Indo-European (Romani) | Med13 | 15 | 16 | 14 | 22 | 10 | 11 | 12 | 14 | 9 | 11 | 18 | 15 | 17 | 20 | 12 | Klaric et al. 2009 |
| Europe | Croatia | Indo-European (Romani) | Med14 | 15 | 16 | 14 | 22 | 10 | 11 | 12 | 14 | 9 | 11 | 18 | 15 | 17 | 20 | 12 | Klaric et al. 2009 |
| Europe | Croatia | Indo-European (Romani) | Med15 | 15 | 16 | 14 | 22 | 10 | 11 | 12 | 14 | 9 | 11 | 18 | 15 | 17 | 20 | 12 | Klaric et al. 2009 |
| Europe | Croatia | Indo-European (Romani) | Med16 | 15 | 16 | 14 | 22 | 10 | 11 | 12 | 14 | 9 | 11 | 18 | 15 | 17 | 20 | 12 | Klaric et al. 2009 |
| Europe | Croatia | Indo-European (Romani) | Med17 | 15 | 16 | 14 | 22 | 10 | 11 | 12 | 14 | 9 | 11 | 18 | 15 | 17 | 20 | 12 | Klaric et al. 2009 |
| Europe | Croatia | Indo-European (Romani) | Med18 | 15 | 16 | 14 | 22 | 10 | 11 | 12 | 14 | 9 | 11 | 18 | 15 | 17 | 20 | 12 | Klaric et al. 2009 |
| Europe | Croatia | Indo-European (Romani) | Med19 | 15 | 16 | 14 | 22 | 10 | 11 | 12 | 14 | 9 | 11 | 18 | 15 | 17 | 20 | 12 | Klaric et al. 2009 |
| Europe | Croatia | Indo-European (Romani) | Med20 | 15 | 16 | 14 | 22 | 10 | 11 | 12 | 14 | 9 | 11 | 18 | 15 | 17 | 20 | 12 | Klaric et al. 2009 |
| Europe | Croatia | Indo-European (Romani) | Med21 | 15 | 16 | 14 | 22 | 10 | 11 | 12 | 14 | 9 | 11 | 18 | 15 | 17 | 20 | 12 | Klaric et al. 2009 |
| Europe | Croatia | Indo-European (Romani) | Med22 | 15 | 16 | 14 | 22 | 10 | 11 | 12 | 14 | 9 | 11 | 18 | 15 | 17 | 20 | 12 | Klaric et al. 2009 |
| Europe | Croatia | Indo-European (Romani) | Med23 | 15 | 16 | 14 | 22 | 10 | 11 | 12 | 14 | 9 | 11 | 18 | 15 | 17 | 20 | 12 | Klaric et al. 2009 |
| Europe | Croatia | Indo-European (Romani) | Med24 | 15 | 16 | 14 | 22 | 10 | 11 | 12 | 14 | 9 | 11 | 18 | 15 | 17 | 20 | 12 | Klaric et al. 2009 |
| Europe | Croatia | Indo-European (Romani) | Med25 | 15 | 16 | 14 | 22 | 10 | 11 | 12 | 14 | 9 | 11 | 18 | 15 | 17 | 20 | 12 | Klaric et al. 2009 |
| Europe | Croatia | Indo-European (Romani) | Med26 | 15 | 16 | 14 | 22 | 10 | 11 | 12 | 14 | 9 | 11 | 18 | 15 | 17 | 20 | 12 | Klaric et al. 2009 |
| Europe | Croatia | Indo-European (Romani) | Med27 | 15 | 16 | 14 | 22 | 10 | 11 | 12 | 14 | 9 | 11 | 18 | 15 | 17 | 20 | 12 | Klaric et al. 2009 |
| Europe | Croatia | Indo-European (Romani) | Med28 | 15 | 16 | 14 | 22 | 10 | 11 | 12 | 14 | 9 | 11 | 18 | 15 | 17 | 20 | 12 | Klaric et al. 2009 |
| East India | Jharkhand | Austroasiatic | ASU20 | 16 | 16 | 12 | 22 | 9 | 12 | 13 | 13 | 11 | 11 | 20 | 16 | 16 | 21 | 12 | Present Study |
| East India | Jharkhand | Austroasiatic | ASU28 | 15 | 15 | 13 | 24 | 10 | 13 | 13 | 14 | 10 | 11 | 18 | 15 | 16 | 21 | 11 | Present Study |
| East India | Jharkhand | Austroasiatic | 338 | 15 | 16 | 13 | 22 | 11 | 11 | 12 | 14 | 9 | 11 | 19 | 16 | 16 | 20 | 12 | Present Study |
| East India | Meghalaya | Austroasiatic | GA12 | 15 | 17 | 13 | 22 | 10 | 13 | 14 | 14 | 10 | 11 | 18 | 15 | 17 | 21 | 11 | Present Study |
| East India | Meghalaya | Austroasiatic | GA14 | 15 | 17 | 13 | 22 | 10 | 13 | 14 | 15 | 10 | 11 | 18 | 15 | 17 | 21 | 11 | Present Study |
| East India | Meghalaya | Austroasiatic | GA17 | 14 | 16 | 13 | 22 | 10 | 13 | 14 | 15 | 10 | 11 | 18 | 15 | 17 | 21 | 11 | Present Study |
| East India | Meghalaya | Austroasiatic | KHP02 | 15 | 16 | 13 | 21 | 10 | 11 | 12 | 14 | 8 | 11 | 19 | 15 | 19 | 20 | 11 | Present Study |
| East India | Orrissa | Austroasiatic | A64 | 15 | 16 | 13 | 22 | 11 | 11 | 13 | 14 | 9 | 11 | 19 | 15 | 18 | 20 | 12 | Present Study |
| East India | Orrissa | Austroasiatic | ORI043 | 14 | 17 | 14 | 21 | 10 | 11 | 13 | 14 | 10 | 12 | 18 | 15 | 16 | 21 | 11 | Present Study |
| East India | Orrissa | Austroasiatic | ORI044 | 14 | 17 | 14 | 21 | 10 | 11 | 13 | 14 | 10 | 12 | 18 | 15 | 16 | 21 | 11 | Present Study |
| East India | Orrissa | Austroasiatic | ORI046 | 14 | 18 | 12 | 22 | 10 | 11 | 12 | 14 | 10 | 11 | 19 | 15 | 16 | 21 | 11 | Present Study |
| East India | Orrissa | Austroasiatic | ORI048 | 14 | 16 | 13 | 22 | 10 | 11 | 12 | 14 | 9 | 11 | 21 | 15 | 18 | 21 | 11 | Present Study |
| East India | Orrissa | Austroasiatic | ORI084 | 16 | 17 | 14 | 21 | 10 | 12 | 13 | 15 | 10 | 11 | 18 | 15 | 16 | 21 | 12 | Present Study |
| East India | Orrissa | Austroasiatic | ORI085 | 14 | 17 | 14 | 21 | 10 | 11 | 13 | 14 | 10 | 12 | 18 | 14 | 16 | 21 | 11 | Present Study |
| North Central India | Madhya Pradesh | Indo-European | SON40 | 16 | 16 | 12 | 24 | 10 | 13 | 12 | 14 | 10 | 11 | 18 | 17 | 16 | 22 | 12 | Present Study |
| North Central India | Madhya Pradesh | Indo-European | SON67 | 15 | 17 | 13 | 24 | 10 | 12 | 12 | 14 | 11 | 11 | 19 | 14 | 17 | 22 | 13 | Present Study |
| North Central India | Madhya Pradesh | Indo-European | BND65 | 14 | 17 | 13 | 24 | 11 | 11 | 13 | 14 | 10 | 11 | 20 | 15 | 17 | 21 | 11 | Present Study |
| North Central India | Madhya Pradesh | Indo-European | BND91 | 16 | 16 | 14 | 22 | 10 | 11 | 11 | 14 | 10 | 12 | 19 | 15 | 16 | 20 | 12 | Present Study |
| North Central India | Madhya Pradesh | Indo-European | BND58 | 14 | 17 | 14 | 23 | 10 | 11 | 13 | 14 | 10 | 11 | 18 | 15 | 17 | 20 | 11 | Present Study |
| North Central India | Madhya Pradesh | Indo-European | RAr82 | 14 | 16 | 12 | 22 | 10 | 12 | 11 | 15 | 10 | 12 | 19 | 16 | 16 | 21 | 12 | Present Study |
| North Central India | Madhya Pradesh | Indo-European | RAR12 | 14 | 16 | 14 | 23 | 10 | 11 | 12 | 15 | 10 | 11 | 20 | 15 | 18 | 22 | 12 | Present Study |
| North Central India | Madhya Pradesh | Indo-European | RAR21 | 15 | 16 | 12 | 24 | 10 | 11 | 11 | 15 | 9 | 12 | 19 | 14 | 17 | 21 | 11 | Present Study |
| North Central India | Madhya Pradesh | Indo-European | RAR30 | 16 | 18 | 14 | 24 | 10 | 11 | 12 | 14 | 11 | 10 | 20 | 15 | 16 | 22 | 12 | Present Study |
| North Central India | Madhya Pradesh | Indo-European | RAR28 | 15 | 16 | 12 | 24 | 10 | 11 | 11 | 15 | 9 | 12 | 19 | 14 | 17 | 21 | 11 | Present Study |
| North Central India | Madhya Pradesh | Indo-European | BAI56 | 16 | 16 | 13 | 22 | 10 | 11 | 11 | 14 | 10 | 11 | 19 | 15 | 16 | 21 | 12 | Present Study |
| North Central India | Madhya Pradesh | Indo-European | BAI60 | 16 | 16 | 13 | 22 | 10 | 11 | 11 | 14 | 10 | 11 | 19 | 15 | 16 | 21 | 12 | Present Study |
| North Central India | Madhya Pradesh | Indo-European | BAI58 | 16 | 16 | 13 | 22 | 10 | 11 | 11 | 14 | 10 | 11 | 19 | 15 | 16 | 21 | 12 | Present Study |
| North Central India | Madhya Pradesh | Indo-European | BAI62 | 16 | 16 | 13 | 22 | 10 | 11 | 11 | 14 | 10 | 11 | 19 | 15 | 16 | 21 | 12 | Present Study |
| North Central India | Madhya Pradesh | Indo-European | BAI66 | 16 | 16 | 13 | 22 | 10 | 11 | 11 | 14 | 10 | 11 | 19 | 14 | 16 | 22 | 12 | Present Study |
| North Central India | Madhya Pradesh | Indo-European | BAI59 | 16 | 16 | 13 | 22 | 10 | 11 | 11 | 14 | 10 | 11 | 19 | 15 | 16 | 21 | 12 | Present Study |
| North Central India | Madhya Pradesh | Indo-European | BAI65 | 16 | 16 | 13 | 22 | 10 | 11 | 11 | 14 | 10 | 11 | 19 | 15 | 16 | 21 | 12 | Present Study |
| North Central India | Madhya Pradesh | Indo-European | BAI63 | 16 | 16 | 13 | 22 | 10 | 11 | 11 | 14 | 10 | 11 | 19 | 15 | 16 | 21 | 12 | Present Study |
| North Central India | Madhya Pradesh | Indo-European | BAI64 | 16 | 16 | 13 | 22 | 10 | 11 | 11 | 14 | 10 | 11 | 19 | 15 | 16 | 21 | 12 | Present Study |
| North Central India | Madhya Pradesh | Indo-European | BAI57 | 16 | 16 | 13 | 22 | 10 | 11 | 11 | 14 | 10 | 11 | 19 | 15 | 16 | 21 | 12 | Present Study |
| North Central India | Madhya Pradesh | Indo-European | Bi12 | 15 | 16 | 12 | 22 | 11 | 13 | 11 | 14 | 9 | 11 | 18 | 15 | 18 | 20 | 12 | Sharma et al. 2012 |
| North Central India | Madhya Pradesh | Indo-European | Bi13 | 15 | 16 | 12 | 22 | 10 | 11 | 11 | 14 | 9 | 11 | 19 | 16 | 18 | 20 | 12 | Sharma et al. 2012 |
| North Central India | Madhya Pradesh | Indo-European | Bi14 | 15 | 16 | 12 | 22 | 11 | 13 | 11 | 14 | 9 | 11 | 18 | 16 | 17 | 20 | 11 | Sharma et al. 2012 |
| North Central India | Madhya Pradesh | Indo-European | Bi15 | 15 | 16 | 12 | 22 | 10 | 11 | 11 | 14 | 9 | 11 | 19 | 16 | 18 | 20 | 12 | Sharma et al. 2012 |
| North Central India | Madhya Pradesh | Indo-European | Bi17 | 15 | 16 | 12 | 22 | 11 | 13 | 11 | 14 | 9 | 11 | 18 | 15 | 18 | 21 | 12 | Sharma et al. 2012 |
| North Central India | Madhya Pradesh | Indo-European | Bi19 | 15 | 16 | 12 | 22 | 10 | 11 | 11 | 14 | 9 | 11 | 19 | 16 | 17 | 21 | 12 | Sharma et al. 2012 |
| North Central India | Madhya Pradesh | Indo-European | Bi20 | 16 | 16 | 12 | 22 | 11 | 13 | 11 | 14 | 9 | 11 | 18 | 16 | 17 | 20 | 11 | Sharma et al. 2012 |
| North Central India | Madhya Pradesh | Indo-European | Bi29 | 15 | 16 | 13 | 22 | 10 | 11 | 11 | 14 | 9 | 11 | 19 | 15 | 19 | 20 | 12 | Sharma et al. 2012 |
| North Central India | Madhya Pradesh | Indo-European | Bi3 | 14 | 16 | 13 | 21 | 10 | 11 | 11 | 14 | 9 | 12 | 20 | 17 | 16 | 20 | 13 | Sharma et al. 2012 |
| North Central India | Madhya Pradesh | Indo-European | Bi30 | 15 | 16 | 13 | 23 | 10 | 11 | 11 | 14 | 9 | 11 | 19 | 15 | 17 | 20 | 11 | Sharma et al. 2012 |
| North Central India | Madhya Pradesh | Indo-European | Bi31 | 14 | 16 | 13 | 21 | 10 | 11 | 11 | 14 | 9 | 12 | 20 | 17 | 16 | 20 | 13 | Sharma et al. 2012 |
| North Central India | Madhya Pradesh | Indo-European | Bi34 | 15 | 17 | 12 | 21 | 9 | 11 | 11 | 13 | 10 | 9 | 19 | 14 | 16 | 22 | 12 | Sharma et al. 2012 |
| North Central India | Madhya Pradesh | Indo-European | Bi25 | 14 | 16 | 13 | 21 | 10 | 11 | 11 | 14 | 9 | 12 | 20 | 17 | 16 | 20 | 13 | Sharma et al. 2012 |
| North Central India | Madhya Pradesh | Indo-European | Bi33 | 14 | 16 | 13 | 21 | 10 | 11 | 11 | 14 | 9 | 12 | 20 | 17 | 16 | 20 | 13 | Sharma et al. 2012 |
| North Central India | Madhya Pradesh | Indo-European | Bi34a | 15 | 16 | 13 | 22 | 10 | 11 | 11 | 14 | 9 | 11 | 19 | 15 | 19 | 20 | 12 | Sharma et al. 2012 |
| North India | Uttar Pradesh | Indo-European | PAL67 | 14 | 16 | 13 | 22 | 10 | 11 | 11 | 14 | 9 | 11 | 19 | 16 | 16 | 20 | 13 | Present Study |
| North India | Uttar Pradesh | Indo-European | PAL77 | 14 | 16 | 13 | 22 | 10 | 11 | 11 | 14 | 9 | 11 | 19 | 16 | 16 | 20 | 11 | Present Study |
| North India | Uttar Pradesh | Indo-European | PAL57 | 13 | 16 | 12 | 22 | 10 | 11 | 11 | 15 | 10 | 11 | 19 | 15 | 17 | 22 | 12 | Present Study |
| North India | Uttar Pradesh | Indo-European | PAL74 | 14 | 16 | 13 | 22 | 10 | 11 | 11 | 14 | 9 | 11 | 19 | 16 | 16 | 20 | 13 | Present Study |
| North India | Uttar Pradesh | Indo-European | PAL63 | 14 | 16 | 13 | 22 | 10 | 11 | 11 | 14 | 9 | 11 | 19 | 16 | 16 | 20 | 13 | Present Study |
| North India | Uttar Pradesh | Indo-European | PAL66 | 14 | 18 | 13 | 22 | 10 | 11 | 11 | 14 | 9 | 11 | 19 | 16 | 16 | 20 | 13 | Present Study |
| North India | Uttar Pradesh | Indo-European | SHI21 | 16 | 17 | 12 | 22 | 10 | 11 | 11 | 14 | 10 | 10 | 20 | 14 | 17 | 22 | 12 | Present Study |
| North India | Uttar Pradesh | Indo-European | SHI38 | 15 | 16 | 12 | 23 | 10 | 12 | 12 | 15 | 10 | 11 | 19 | 15 | 16 | 19 | 12 | Present Study |
| North India | Uttar Pradesh | Indo-European | SHI46 | 15 | 18 | 13 | 22 | 10 | 13 | 11 | 14 | 10 | 11 | 20 | 16 | 18 | 21 | 12 | Present Study |
| North India | Uttar Pradesh | Indo-European | Th1 | 15 | 16 | 14 | 22 | 10 | 11 | 11 | 14 | 10 | 11 | 19 | 16 | 18 | 19 | 12 | Present Study |
| North India | Uttar Pradesh | Indo-European | Th2 | 15 | 15 | 12 | 23 | 10 | 11 | 11 | 15 | 11 | 12 | 20 | 16 | 18 | 19 | 12 | Present Study |
| North India | Uttar Pradesh | Indo-European | Th3 | 15 | 17 | 13 | 24 | 11 | 11 | 12 | 14 | 11 | 10 | 20 | 15 | 17 | 18 | 12 | Present Study |
| North India | Uttar Pradesh | Indo-European | Th4 | 15 | 16 | 14 | 22 | 10 | 11 | 11 | 14 | 10 | 12 | 19 | 15 | 16 | 18 | 12 | Present Study |
| North India | Uttar Pradesh | Indo-European | Th5 | 15 | 16 | 13 | 22 | 11 | 11 | 11 | 14 | 10 | 12 | 19 | 15 | 18 | 19 | 13 | Present Study |
| North India | Uttar Pradesh | Indo-European | Th6 | 15 | 16 | 13 | 22 | 10 | 11 | 11 | 14 | 10 | 11 | 19 | 16 | 17 | 18 | 11 | Present Study |
| North India | Uttar Pradesh | Indo-European | Th7 | 15 | 16 | 13 | 22 | 11 | 11 | 11 | 14 | 10 | 11 | 19 | 15 | 18 | 19 | 13 | Present Study |
| North India | Uttar Pradesh | Indo-European | Th8 | 16 | 17 | 12 | 24 | 11 | 11 | 12 | 14 | 10 | 10 | 20 | 15 | 17 | 18 | 13 | Present Study |
| North India | Uttar Pradesh | Indo-European | Th9 | 16 | 16 | 13 | 24 | 10 | 11 | 12 | 15 | 10 | 12 | 21 | 15 | 17 | 18 | 13 | Present Study |
| North India | Uttar Pradesh | Indo-European | BR020 | 15 | 17 | 13 | 23 | 10 | 11 | 11 | 14 | 9 | 12 | 19 | 16 | 16 | 20 | 12 | Present Study |
| North India | Uttar Pradesh | Indo-European | BR021 | 16 | 17 | 14 | 22 | 11 | 12 | 12 | 14 | 9 | 11 | 19 | 15 | 17 | 21 | 11 | Present Study |
| North India | Uttar Pradesh | Indo-European | GBR021 | 14 | 17 | 13 | 22 | 11 | 11 | 12 | 14 | 9 | 12 | 19 | 15 | 17 | 21 | 11 | Present Study |
| North India | Uttar Pradesh | Indo-European | GBR022 | 16 | 17 | 14 | 22 | 11 | 11 | 12 | 14 | 10 | 12 | 19 | 15 | 16 | 21 | 11 | Present Study |
| North India | Uttar Pradesh | Indo-European | GBR023 | 16 | 17 | 14 | 22 | 11 | 11 | 12 | 14 | 9 | 12 | 19 | 15 | 17 | 21 | 11 | Present Study |
| North India | Uttar Pradesh | Indo-European | GBR024 | 15 | 17 | 13 | 22 | 11 | 11 | 12 | 14 | 9 | 12 | 19 | 14 | 17 | 21 | 11 | Present Study |
| North India | Uttar Pradesh | Indo-European | GBR025 | 16 | 17 | 14 | 23 | 11 | 11 | 12 | 13 | 10 | 12 | 19 | 15 | 17 | 21 | 12 | Present Study |
| North India | Uttar Pradesh | Indo-European | GBR026 | 16 | 17 | 13 | 22 | 11 | 12 | 12 | 14 | 9 | 11 | 19 | 15 | 17 | 21 | 11 | Present Study |
| Northwest India | Gujrat | Indo-European | St1 | 15 | 16 | 14 | 22 | 10 | 11 | 12 | 14 | 9 | 10 | 19 | 15 | 17 | 20 | 12 | Present Study |
| Northwest India | Gujrat | Indo-European | St22 | 15 | 16 | 14 | 22 | 10 | 11 | 12 | 14 | 9 | 10 | 19 | 15 | 17 | 20 | 12 | Present Study |
| Northwest India | Gujrat | Indo-European | St31 | 15 | 16 | 14 | 22 | 10 | 11 | 12 | 14 | 9 | 10 | 19 | 15 | 17 | 20 | 12 | Present Study |
| Northwest India | Gujrat | Indo-European | St14 | 15 | 16 | 14 | 22 | 10 | 11 | 12 | 14 | 9 | 11 | 19 | 16 | 17 | 20 | 12 | Present Study |
| Northwest India | Gujrat | Indo-European | St51 | 15 | 15 | 14 | 22 | 10 | 11 | 12 | 14 | 9 | 11 | 19 | 15 | 17 | 20 | 13 | Present Study |
| Northwest India | Gujrat | Indo-European | St26 | 15 | 16 | 14 | 22 | 10 | 11 | 12 | 14 | 9 | 11 | 19 | 15 | 17 | 20 | 12 | Present Study |
| Northwest India | Gujrat | Indo-European | St67 | 15 | 16 | 14 | 22 | 10 | 11 | 12 | 14 | 9 | 10 | 19 | 15 | 17 | 21 | 12 | Present Study |
| Northwest India | Gujrat | Indo-European | St38 | 14 | 16 | 14 | 22 | 10 | 11 | 12 | 14 | 9 | 10 | 19 | 16 | 16 | 20 | 12 | Present Study |
| Northwest India | Gujrat | Indo-European | St29 | 14 | 16 | 14 | 22 | 10 | 11 | 13 | 14 | 9 | 10 | 19 | 17 | 17 | 21 | 12 | Present Study |
| Northwest India | Gujrat | Indo-European | St101 | 14 | 16 | 14 | 22 | 10 | 11 | 12 | 14 | 9 | 10 | 19 | 16 | 17 | 20 | 12 | Present Study |
| Northwest India | Gujrat | Indo-European | St11 | 14 | 16 | 14 | 22 | 10 | 11 | 12 | 14 | 9 | 10 | 19 | 15 | 17 | 20 | 13 | Present Study |
| Northwest India | Gujrat | Indo-European | g92 | 14 | 17 | 12 | 21 | 9 | 11 | 12 | 13 | 9 | 10 | 19 | 16 | 17 | 20 | 11 | Present Study |
| Northwest India | Gujrat | Indo-European | St98 | 15 | 16 | 14 | 21 | 10 | 11 | 13 | 14 | 9 | 11 | 19 | 16 | 17 | 20 | 12 | Present Study |
| Northwest India | Gujrat | Indo-European | g31 | 15 | 17 | 14 | 22 | 11 | 11 | 11 | 14 | 9 | 11 | 19 | 16 | 18 | 21 | 11 | Present Study |
| Northwest India | Gujrat | Indo-European | g34 | 15 | 17 | 14 | 22 | 11 | 11 | 11 | 14 | 9 | 11 | 19 | 16 | 18 | 21 | 11 | Present Study |
| Northwest India | Gujrat | Indo-European | g39 | 15 | 17 | 14 | 22 | 11 | 11 | 11 | 14 | 9 | 10 | 19 | 16 | 18 | 21 | 11 | Present Study |
| Northwest India | Haryana | Indo-European | KAM72 | 15 | 17 | 13 | 24 | 10 | 11 | 11 | 15 | 10 | 11 | 19 | 15 | 18 | 21 | 11 | Present Study |
| Northwest India | Haryana | Indo-European | KAM67 | 16 | 17 | 13 | 22 | 10 | 11 | 11 | 14 | 8 | 11 | 19 | 15 | 17 | 20 | 12 | Present Study |
| Northwest India | Haryana | Indo-European | KAM74 | 15 | 18 | 13 | 24 | 10 | 11 | 11 | 14 | 10 | 11 | 18 | 16 | 16 | 20 | 11 | Present Study |
| Northwest India | Haryana | Indo-European | KAM71 | 15 | 17 | 13 | 24 | 10 | 11 | 11 | 15 | 10 | 11 | 19 | 15 | 18 | 21 | 11 | Present Study |
| Northwest India | Haryana | Indo-European | KAM76 | 15 | 17 | 13 | 24 | 10 | 11 | 11 | 15 | 10 | 11 | 19 | 15 | 18 | 21 | 11 | Present Study |
| Northwest India | Haryana | Indo-European | sc36 | 15 | 16 | 14 | 21 | 10 | 11 | 12 | 14 | 9 | 11 | 19 | 15 | 17 | 21 | 12 | Present Study |
| Northwest India | Haryana | Indo-European | b65 | 13 | 16 | 13 | 22 | 10 | 11 | 12 | 14 | 10 | 11 | 19 | 15 | 17 | 20 | 13 | Present Study |
| Northwest India | Haryana | Indo-European | b66 | 13 | 18 | 12 | 22 | 10 | 11 | 13 | 14 | 10 | 11 | 19 | 15 | 17 | 22 | 13 | Present Study |
| Northwest India | Haryana | Indo-European | b91 | 13 | 16 | 13 | 22 | 10 | 11 | 12 | 14 | 10 | 11 | 19 | 16 | 17 | 20 | 13 | Present Study |
| Northwest India | Haryana | Indo-European | b59 | 13 | 16 | 13 | 22 | 10 | 11 | 12 | 13 | 10 | 11 | 19 | 15 | 16 | 20 | 13 | Present Study |
| Northwest India | Haryana | Indo-European | c103 | 13 | 17 | 13 | 22 | 10 | 11 | 12 | 13 | 10 | 11 | 19 | 15 | 17 | 21 | 13 | Present Study |
| Northwest India | Haryana | Indo-European | c131 | 13 | 16 | 13 | 22 | 11 | 11 | 12 | 14 | 9 | 12 | 19 | 16 | 17 | 21 | 13 | Present Study |
| Northwest India | Haryana | Indo-European | c146 | 13 | 17 | 13 | 22 | 10 | 11 | 12 | 14 | 10 | 11 | 19 | 15 | 17 | 20 | 13 | Present Study |
| Northwest India | Haryana | Indo-European | c150 | 13 | 17 | 13 | 22 | 10 | 11 | 12 | 14 | 9 | 11 | 19 | 15 | 16 | 20 | 12 | Present Study |
| Northwest India | Haryana | Indo-European | p172 | 13 | 17 | 13 | 21 | 10 | 11 | 12 | 14 | 10 | 11 | 19 | 15 | 17 | 21 | 13 | Present Study |
| Northwest India | Haryana | Indo-European | p173 | 13 | 17 | 13 | 22 | 10 | 11 | 12 | 14 | 10 | 11 | 19 | 15 | 18 | 20 | 13 | Present Study |
| Northwest India | Haryana | Indo-European | p174 | 13 | 17 | 14 | 22 | 9 | 11 | 13 | 14 | 11 | 12 | 19 | 15 | 17 | 22 | 13 | Present Study |
| Northwest India | Haryana | Indo-European | p197 | 13 | 18 | 14 | 22 | 11 | 11 | 13 | 14 | 10 | 12 | 19 | 16 | 16 | 19 | 11 | Present Study |
| Northwest India | Haryana | Indo-European | p171 | 13 | 16 | 13 | 22 | 10 | 11 | 12 | 14 | 10 | 12 | 18 | 16 | 17 | 20 | 13 | Present Study |
| Northwest India | Haryana | Indo-European | sc17 | 13 | 16 | 13 | 22 | 10 | 11 | 12 | 14 | 9 | 11 | 19 | 15 | 16 | 21 | 12 | Present Study |
| Northwest India | Haryana | Indo-European | sc18 | 15 | 17 | 14 | 22 | 10 | 11 | 12 | 14 | 9 | 13 | 19 | 14 | 16 | 20 | 11 | Present Study |
| Northwest India | Haryana | Indo-European | sc27 | 13 | 16 | 13 | 22 | 10 | 11 | 12 | 14 | 10 | 11 | 19 | 15 | 18 | 20 | 13 | Present Study |
| Northwest India | Haryana | Indo-European | sc31 | 13 | 16 | 13 | 22 | 11 | 11 | 12 | 14 | 9 | 11 | 20 | 16 | 16 | 20 | 12 | Present Study |
| Northwest India | Haryana | Indo-European | sc44 | 13 | 17 | 13 | 22 | 10 | 11 | 12 | 14 | 9 | 11 | 19 | 15 | 17 | 20 | 12 | Present Study |
| Northwest India | Haryana | Indo-European | sc15 | 14 | 17 | 13 | 22 | 10 | 11 | 12 | 14 | 9 | 12 | 19 | 16 | 19 | 20 | 12 | Present Study |
| Northwest India | Haryana | Indo-European | sc49 | 14 | 17 | 13 | 22 | 10 | 11 | 12 | 14 | 9 | 11 | 19 | 14 | 16 | 20 | 12 | Present Study |
| Northwest India | Haryana | Indo-European | sc16 | 14 | 17 | 14 | 22 | 11 | 11 | 13 | 14 | 9 | 12 | 19 | 15 | 18 | 21 | 12 | Present Study |
| Northwest India | Rajasthan | Indo-European | Sc1 | 15 | 16 | 13 | 22 | 10 | 11 | 12 | 14 | 9 | 11 | 19 | 15 | 16 | 20 | 12 | Present Study |
| Northwest India | Rajasthan | Indo-European | Sc2 | 14 | 15 | 14 | 22 | 10 | 11 | 12 | 14 | 9 | 11 | 20 | 15 | 19 | 22 | 12 | Present Study |
| Northwest India | Rajasthan | Indo-European | Sc3 | 15 | 16 | 13 | 22 | 10 | 11 | 12 | 14 | 9 | 11 | 19 | 15 | 16 | 20 | 11 | Present Study |
| Northwest India | Rajasthan | Indo-European | St22 | 16 | 15 | 14 | 21 | 11 | 11 | 13 | 15 | 10 | 10 | 19 | 15 | 17 | 22 | 11 | Present Study |
| Northwest India | Rajasthan | Indo-European | St13 | 14 | 15 | 14 | 22 | 10 | 11 | 11 | 13 | 9 | 11 | 19 | 15 | 18 | 21 | 12 | Present Study |
| Northwest India | Rajasthan | Indo-European | St14 | 15 | 16 | 14 | 22 | 10 | 11 | 12 | 14 | 9 | 11 | 19 | 16 | 17 | 20 | 12 | Present Study |
| Northwest India | Rajasthan | Indo-European | St25 | 15 | 16 | 14 | 22 | 10 | 11 | 12 | 14 | 9 | 11 | 19 | 15 | 19 | 21 | 12 | Present Study |
| Northwest India | Rajasthan | Indo-European | St16 | 15 | 16 | 14 | 22 | 10 | 11 | 12 | 14 | 9 | 11 | 19 | 16 | 17 | 20 | 12 | Present Study |
| Northwest India | Rajasthan | Indo-European | Sc34 | 14 | 17 | 14 | 23 | 10 | 11 | 13 | 14 | 9 | 11 | 18 | 16 | 16 | 20 | 12 | Present Study |
| Northwest India | Rajasthan | Indo-European | Sc4 | 15 | 16 | 14 | 22 | 10 | 11 | 12 | 14 | 9 | 12 | 19 | 15 | 18 | 21 | 12 | Present Study |
| Northwest India | Rajasthan | Indo-European | j74 | 14 | 17 | 13 | 22 | 10 | 11 | 12 | 14 | 10 | 11 | 19 | 16 | 17 | 20 | 12 | Present Study |
| Northwest India | Rajasthan | Indo-European | j10 | 15 | 16 | 13 | 22 | 10 | 11 | 12 | 14 | 9 | 11 | 19 | 16 | 18 | 20 | 11 | Present Study |
| Northwest India | Rajasthan | Indo-European | j40 | 15 | 17 | 13 | 23 | 10 | 11 | 13 | 14 | 10 | 10 | 20 | 15 | 18 | 22 | 13 | Present Study |
| Northwest India | Rajasthan | Indo-European | j42 | 14 | 18 | 14 | 22 | 10 | 11 | 13 | 14 | 9 | 11 | 18 | 16 | 17 | 20 | 13 | Present Study |
| Northwest India | Rajasthan | Indo-European | j16 | 15 | 16 | 14 | 22 | 10 | 11 | 12 | 14 | 9 | 11 | 19 | 15 | 18 | 21 | 13 | Present Study |
| Northwest India | Rajasthan | Indo-European | j65 | 15 | 16 | 13 | 22 | 10 | 11 | 13 | 14 | 9 | 11 | 19 | 16 | 18 | 20 | 13 | Present Study |
| Northwest India | Rajasthan | Indo-European | j27 | 14 | 17 | 14 | 22 | 9 | 11 | 13 | 14 | 9 | 11 | 18 | 16 | 16 | 20 | 11 | Present Study |
| Northwest India | Rajasthan | Indo-European | j69 | 13 | 18 | 13 | 22 | 9 | 11 | 12 | 13 | 9 | 10 | 18 | 16 | 16 | 21 | 12 | Present Study |
| Northwest India | Rajasthan | Indo-European | j71 | 14 | 17 | 12 | 21 | 9 | 11 | 13 | 13 | 9 | 11 | 19 | 15 | 17 | 20 | 12 | Present Study |
| Northwest India | Rajasthan | Indo-European | j37 | 16 | 16 | 14 | 22 | 10 | 11 | 12 | 14 | 10 | 12 | 19 | 16 | 16 | 21 | 13 | Present Study |
| Northwest India | Rajasthan | Indo-European | j73 | 15 | 17 | 14 | 23 | 10 | 11 | 12 | 14 | 9 | 11 | 19 | 16 | 16 | 21 | 12 | Present Study |
| Europe | Portugal | Indo-European (Romani) | Por1 | 15 | 16 | 14 | 22 | 10 | 11 | 12 | 14 | 9 | 11 | 19 | 15 | 16 | 20 | 12 | Gusmao et al. 2008 |
| Europe | Portugal | Indo-European (Romani) | Por2 | 15 | 16 | 14 | 22 | 10 | 11 | 12 | 14 | 9 | 11 | 19 | 15 | 17 | 20 | 12 | Gusmao et al. 2008 |
| Europe | Portugal | Indo-European (Romani) | Por3 | 15 | 16 | 14 | 22 | 10 | 11 | 12 | 14 | 9 | 11 | 19 | 15 | 17 | 20 | 12 | Gusmao et al. 2008 |
| Europe | Portugal | Indo-European (Romani) | Por4 | 15 | 16 | 14 | 22 | 10 | 11 | 12 | 14 | 9 | 11 | 19 | 15 | 17 | 20 | 12 | Gusmao et al. 2008 |
| Europe | Portugal | Indo-European (Romani) | Por5 | 15 | 16 | 14 | 22 | 10 | 11 | 12 | 14 | 9 | 11 | 19 | 15 | 17 | 20 | 12 | Gusmao et al. 2008 |
| Europe | Portugal | Indo-European (Romani) | Por6 | 15 | 16 | 14 | 22 | 10 | 11 | 12 | 14 | 9 | 11 | 19 | 15 | 17 | 20 | 12 | Gusmao et al. 2008 |
| Europe | Portugal | Indo-European (Romani) | Por7 | 15 | 16 | 14 | 22 | 10 | 11 | 12 | 14 | 9 | 11 | 19 | 15 | 17 | 20 | 12 | Gusmao et al. 2008 |
| Europe | Portugal | Indo-European (Romani) | Por8 | 15 | 16 | 14 | 22 | 10 | 11 | 12 | 14 | 9 | 11 | 19 | 15 | 18 | 20 | 12 | Gusmao et al. 2008 |
| Europe | Portugal | Indo-European (Romani) | Por9 | 15 | 16 | 14 | 22 | 10 | 11 | 12 | 14 | 9 | 11 | 19 | 15 | 18 | 20 | 12 | Gusmao et al. 2008 |
| Europe | Portugal | Indo-European (Romani) | Por10 | 15 | 16 | 14 | 22 | 10 | 11 | 12 | 14 | 9 | 11 | 19 | 15 | 19 | 20 | 12 | Gusmao et al. 2008 |
| Europe | Portugal | Indo-European (Romani) | Por11 | 15 | 16 | 14 | 22 | 10 | 11 | 12 | 14 | 9 | 11 | 19 | 15 | 19 | 20 | 12 | Gusmao et al. 2008 |
| Europe | Portugal | Indo-European (Romani) | Por12 | 15 | 16 | 14 | 22 | 10 | 11 | 12 | 14 | 9 | 11 | 19 | 15 | 19 | 20 | 12 | Gusmao et al. 2008 |
| Europe | Portugal | Indo-European (Romani) | Por13 | 15 | 16 | 14 | 22 | 10 | 11 | 12 | 14 | 9 | 11 | 19 | 15 | 19 | 21 | 12 | Gusmao et al. 2008 |
| Europe | Portugal | Indo-European (Romani) | Por14 | 15 | 16 | 14 | 22 | 10 | 11 | 12 | 14 | 9 | 11 | 19 | 16 | 17 | 20 | 12 | Gusmao et al. 2008 |
| Europe | Portugal | Indo-European (Romani) | Por15 | 15 | 16 | 14 | 22 | 10 | 11 | 12 | 14 | 9 | 12 | 19 | 15 | 17 | 20 | 11 | Gusmao et al. 2008 |
| Europe | Portugal | Indo-European (Romani) | Por16 | 15 | 16 | 14 | 22 | 10 | 11 | 12 | 14 | 9 | 12 | 19 | 15 | 18 | 20 | 13 | Gusmao et al. 2008 |
| Europe | Portugal | Indo-European (Romani) | Por17 | 15 | 16 | 14 | 22 | 10 | 11 | 12 | 14 | 9 | 12 | 19 | 15 | 18 | 20 | 13 | Gusmao et al. 2008 |
| Europe | Portugal | Indo-European (Romani) | Por18 | 15 | 16 | 14 | 22 | 10 | 11 | 12 | 14 | 9 | 11 | 19 | 15 | 17 | 20 | 12 | Gusmao et al. 2008 |
| Europe | Portugal | Indo-European (Romani) | Por19 | 15 | 16 | 14 | 22 | 10 | 11 | 12 | 14 | 9 | 11 | 19 | 15 | 17 | 20 | 12 | Gusmao et al. 2008 |
| Europe | Portugal | Indo-European (Romani) | Por20 | 15 | 16 | 14 | 22 | 10 | 11 | 12 | 14 | 9 | 11 | 19 | 15 | 18 | 20 | 12 | Gusmao et al. 2008 |
| Europe | Portugal | Indo-European (Romani) | Por21 | 15 | 16 | 14 | 22 | 10 | 11 | 12 | 14 | 9 | 12 | 19 | 15 | 17 | 20 | 11 | Gusmao et al. 2008 |
| Europe | Serbia | Indo-European (Romani) | Bel1 | 15 | 16 | 15 | 22 | 10 | 11 | 12 | 14 | 9 | 11 | 19 | 15 | 18 | 20 | 12 | Maria Regueiro et al.2011 |
| Europe | Serbia | Indo-European (Romani) | Bel2 | 15 | 16 | 15 | 22 | 10 | 11 | 12 | 14 | 9 | 11 | 19 | 15 | 18 | 20 | 12 | Maria Regueiro et al.2011 |
| Europe | Serbia | Indo-European (Romani) | Bel3 | 15 | 16 | 14 | 22 | 10 | 11 | 12 | 14 | 9 | 11 | 19 | 15 | 18 | 20 | 12 | Maria Regueiro et al.2011 |
| Europe | Serbia | Indo-European (Romani) | Bel4 | 15 | 16 | 14 | 22 | 10 | 11 | 12 | 14 | 9 | 11 | 19 | 15 | 18 | 20 | 12 | Maria Regueiro et al.2011 |
| Europe | Serbia | Indo-European (Romani) | Bel5 | 15 | 16 | 14 | 22 | 10 | 11 | 12 | 14 | 9 | 11 | 19 | 15 | 18 | 20 | 12 | Maria Regueiro et al.2011 |
| Europe | Serbia | Indo-European (Romani) | Bel6 | 15 | 16 | 14 | 22 | 10 | 11 | 12 | 14 | 9 | 11 | 19 | 15 | 19 | 20 | 12 | Maria Regueiro et al.2011 |
| Europe | Serbia | Indo-European (Romani) | Bel7 | 15 | 16 | 14 | 22 | 10 | 11 | 12 | 14 | 9 | 11 | 19 | 14 | 18 | 20 | 12 | Maria Regueiro et al.2011 |
| Europe | Serbia | Indo-European (Romani) | Bel8 | 15 | 16 | 14 | 22 | 10 | 11 | 12 | 14 | 9 | 11 | 19 | 15 | 19 | 20 | 12 | Maria Regueiro et al.2011 |
| Europe | Serbia | Indo-European (Romani) | Bel9 | 15 | 16 | 14 | 22 | 10 | 11 | 12 | 14 | 9 | 11 | 19 | 15 | 19 | 20 | 12 | Maria Regueiro et al.2011 |
| Europe | Serbia | Indo-European (Romani) | Bel10 | 15 | 16 | 14 | 22 | 10 | 11 | 12 | 14 | 9 | 11 | 19 | 15 | 18 | 20 | 12 | Maria Regueiro et al.2011 |
| Europe | Serbia | Indo-European (Romani) | Bel11 | 15 | 16 | 14 | 22 | 10 | 11 | 12 | 14 | 9 | 11 | 19 | 15 | 17 | 20 | 12 | Maria Regueiro et al.2011 |
| Europe | Serbia | Indo-European (Romani) | Bel12 | 15 | 16 | 14 | 22 | 10 | 11 | 12 | 14 | 9 | 11 | 19 | 15 | 17 | 20 | 12 | Maria Regueiro et al.2011 |
| Europe | Serbia | Indo-European (Romani) | Bel13 | 15 | 17 | 14 | 22 | 10 | 11 | 12 | 14 | 9 | 11 | 19 | 15 | 18 | 20 | 12 | Maria Regueiro et al.2011 |
| Europe | Serbia | Indo-European (Romani) | Bel14 | 15 | 16 | 14 | 22 | 10 | 11 | 12 | 14 | 9 | 11 | 19 | 15 | 18 | 20 | 12 | Maria Regueiro et al.2011 |
| Europe | Serbia | Indo-European (Romani) | Bel15 | 15 | 16 | 14 | 22 | 10 | 11 | 12 | 14 | 9 | 13 | 19 | 15 | 18 | 20 | 12 | Maria Regueiro et al.2011 |
| Europe | Serbia | Indo-European (Romani) | Bel16 | 15 | 16 | 14 | 22 | 11 | 11 | 12 | 14 | 9 | 12 | 19 | 15 | 18 | 20 | 12 | Maria Regueiro et al.2011 |
| Europe | Serbia | Indo-European (Romani) | Bel17 | 15 | 16 | 14 | 22 | 10 | 12 | 12 | 14 | 9 | 11 | 19 | 15 | 19 | 20 | 12 | Maria Regueiro et al.2011 |
| Europe | Serbia | Indo-European (Romani) | Bel18 | 15 | 16 | 14 | 22 | 10 | 12 | 12 | 14 | 9 | 11 | 19 | 15 | 18 | 20 | 12 | Maria Regueiro et al.2011 |
| Europe | Serbia | Indo-European (Romani) | Kos1 | 15 | 16 | 15 | 22 | 10 | 11 | 12 | 14 | 9 | 11 | 19 | 15 | 18 | 20 | 12 | Maria Regueiro et al.2011 |
| Europe | Serbia | Indo-European (Romani) | Kos2 | 15 | 16 | 15 | 22 | 10 | 11 | 12 | 14 | 9 | 11 | 19 | 15 | 18 | 20 | 12 | Maria Regueiro et al.2011 |
| Europe | Serbia | Indo-European (Romani) | Kos3 | 15 | 16 | 15 | 22 | 10 | 11 | 12 | 14 | 9 | 11 | 19 | 15 | 18 | 20 | 12 | Maria Regueiro et al.2011 |
| Europe | Serbia | Indo-European (Romani) | Kos4 | 15 | 16 | 15 | 22 | 10 | 11 | 12 | 14 | 9 | 11 | 19 | 15 | 18 | 20 | 12 | Maria Regueiro et al.2011 |
| Europe | Serbia | Indo-European (Romani) | Kos5 | 15 | 16 | 15 | 22 | 10 | 11 | 12 | 14 | 9 | 11 | 19 | 15 | 18 | 20 | 12 | Maria Regueiro et al.2011 |
| Europe | Serbia | Indo-European (Romani) | Kos6 | 15 | 16 | 15 | 22 | 10 | 11 | 12 | 14 | 9 | 11 | 19 | 15 | 18 | 20 | 12 | Maria Regueiro et al.2011 |
| Europe | Serbia | Indo-European (Romani) | Kos7 | 15 | 16 | 14 | 22 | 10 | 11 | 12 | 14 | 9 | 11 | 19 | 15 | 13 | 20 | 12 | Maria Regueiro et al.2011 |
| Europe | Serbia | Indo-European (Romani) | Kos8 | 15 | 16 | 14 | 22 | 10 | 11 | 12 | 14 | 9 | 11 | 19 | 15 | 18 | 20 | 12 | Maria Regueiro et al.2011 |
| Europe | Serbia | Indo-European (Romani) | Kos9 | 15 | 16 | 14 | 22 | 10 | 11 | 12 | 14 | 9 | 11 | 19 | 15 | 18 | 20 | 12 | Maria Regueiro et al.2011 |
| Europe | Serbia | Indo-European (Romani) | Kos10 | 15 | 16 | 14 | 22 | 10 | 11 | 12 | 14 | 9 | 11 | 19 | 15 | 18 | 20 | 12 | Maria Regueiro et al.2011 |
| Europe | Serbia | Indo-European (Romani) | Kos11 | 15 | 16 | 14 | 22 | 10 | 11 | 12 | 14 | 9 | 11 | 19 | 15 | 18 | 20 | 12 | Maria Regueiro et al.2011 |
| Europe | Serbia | Indo-European (Romani) | Kos12 | 15 | 16 | 14 | 22 | 10 | 11 | 12 | 14 | 9 | 11 | 19 | 15 | 18 | 20 | 12 | Maria Regueiro et al.2011 |
| Europe | Serbia | Indo-European (Romani) | Kos13 | 15 | 16 | 14 | 22 | 10 | 11 | 12 | 14 | 9 | 11 | 19 | 15 | 18 | 20 | 12 | Maria Regueiro et al.2011 |
| Europe | Serbia | Indo-European (Romani) | Kos14 | 15 | 16 | 14 | 22 | 10 | 11 | 12 | 14 | 9 | 11 | 19 | 15 | 18 | 20 | 12 | Maria Regueiro et al.2011 |
| Europe | Serbia | Indo-European (Romani) | Kos15 | 15 | 16 | 14 | 22 | 10 | 11 | 12 | 14 | 9 | 11 | 19 | 15 | 18 | 19 | 12 | Maria Regueiro et al.2011 |
| Europe | Serbia | Indo-European (Romani) | Kos16 | 15 | 16 | 14 | 22 | 10 | 11 | 12 | 14 | 9 | 12 | 19 | 15 | 18 | 20 | 12 | Maria Regueiro et al.2011 |
| Europe | Serbia | Indo-European (Romani) | Kos17 | 15 | 16 | 13 | 22 | 10 | 11 | 12 | 14 | 9 | 11 | 19 | 15 | 18 | 20 | 12 | Maria Regueiro et al.2011 |
| Europe | Serbia | Indo-European (Romani) | Kos18 | 15 | 16 | 14 | 22 | 10 | 11 | 12 | 14 | 9 | 12 | 19 | 15 | 18 | 20 | 12 | Maria Regueiro et al.2011 |
| Europe | Serbia | Indo-European (Romani) | Kos19 | 15 | 16 | 14 | 22 | 10 | 11 | 12 | 14 | 9 | 12 | 19 | 15 | 18 | 20 | 12 | Maria Regueiro et al.2011 |
| Europe | Serbia | Indo-European (Romani) | Kos20 | 15 | 16 | 14 | 22 | 10 | 11 | 12 | 14 | 9 | 11 | 19 | 15 | 17 | 20 | 12 | Maria Regueiro et al.2011 |
| Europe | Serbia | Indo-European (Romani) | Kos21 | 15 | 16 | 14 | 22 | 10 | 11 | 12 | 14 | 9 | 11 | 19 | 16 | 18 | 20 | 12 | Maria Regueiro et al.2011 |
| Europe | Serbia | Indo-European (Romani) | Kos22 | 15 | 16 | 15 | 22 | 10 | 11 | 12 | 14 | 9 | 11 | 19 | 15 | 17 | 20 | 12 | Maria Regueiro et al.2011 |
| Europe | Serbia | Indo-European (Romani) | Voj1 | 15 | 16 | 14 | 22 | 10 | 11 | 12 | 14 | 9 | 11 | 18 | 15 | 16 | 20 | 12 | Maria Regueiro et al.2011 |
| South Central India | Chattishgarh | Indo-European | GHA2 | 15 | 17 | 13 | 22 | 11 | 11 | 11 | 14 | 9 | 12 | 19 | 17 | 17 | 20 | 12 | Present Study |
| South Central India | Chattishgarh | Indo-European | GHA8 | 16 | 16 | 12 | 23 | 10 | 11 | 12 | 14 | 11 | 11 | 20 | 15 | 16 | 20 | 12 | Present Study |
| South Central India | Chattishgarh | Indo-European | GHA10 | 16 | 17 | 12 | 23 | 10 | 12 | 12 | 14 | 11 | 11 | 20 | 15 | 16 | 20 | 12 | Present Study |
| South Central India | Chattishgarh | Austroasiatic | KH02 | 15 | 16 | 13 | 22 | 10 | 11 | 13 | 14 | 9 | 13 | 20 | 16 | 17 | 20 | 13 | Present Study |
| South Central India | Chattishgarh | Austroasiatic | KH03 | 15 | 17 | 13 | 22 | 10 | 12 | 12 | 14 | 9 | 13 | 20 | 15 | 17 | 20 | 13 | Present Study |
| South Central India | Chattishgarh | Austroasiatic | KH04 | 15 | 16 | 13 | 22 | 10 | 11 | 13 | 14 | 9 | 13 | 20 | 16 | 17 | 20 | 13 | Present Study |
| South Central India | Chattishgarh | Austroasiatic | KH05 | 15 | 16 | 13 | 22 | 10 | 11 | 12 | 14 | 9 | 13 | 20 | 16 | 17 | 20 | 10 | Present Study |
| South Central India | Chattishgarh | Austroasiatic | KH08 | 15 | 16 | 13 | 22 | 10 | 11 | 13 | 14 | 9 | 10 | 20 | 16 | 17 | 20 | 10 | Present Study |
| South Central India | Chattishgarh | Austroasiatic | KH10 | 15 | 16 | 13 | 22 | 10 | 11 | 12 | 15 | 9 | 13 | 20 | 16 | 17 | 20 | 13 | Present Study |
| South Central India | Chattishgarh | Austroasiatic | KH17 | 15 | 16 | 13 | 22 | 10 | 11 | 13 | 14 | 9 | 13 | 20 | 16 | 17 | 20 | 13 | Present Study |
| South Central India | Chattishgarh | Austroasiatic | KH20 | 15 | 17 | 14 | 21 | 10 | 11 | 14 | 15 | 10 | 10 | 19 | 15 | 17 | 22 | 11 | Present Study |
| South Central India | Chattishgarh | Austroasiatic | KH21 | 15 | 17 | 13 | 22 | 10 | 12 | 13 | 14 | 9 | 13 | 20 | 16 | 17 | 20 | 12 | Present Study |
| South Central India | Chattishgarh | Austroasiatic | KH24 | 15 | 16 | 13 | 22 | 10 | 11 | 12 | 15 | 9 | 13 | 20 | 16 | 17 | 20 | 13 | Present Study |
| South Central India | Chattishgarh | Austroasiatic | KH25 | 15 | 16 | 13 | 22 | 10 | 11 | 12 | 15 | 9 | 13 | 21 | 16 | 17 | 20 | 13 | Present Study |
| South Central India | Chattishgarh | Austroasiatic | KH27 | 16 | 16 | 13 | 22 | 10 | 11 | 12 | 14 | 9 | 11 | 19 | 14 | 17 | 19 | 12 | Present Study |
| South Central India | Madhya Pradesh | Dravidian | PAN52 | 14 | 16 | 12 | 23 | 10 | 11 | 12 | 14 | 9 | 11 | 21 | 15 | 16 | 21 | 12 | Present Study |
| South Central India | Madhya Pradesh | Dravidian | PAN54 | 15 | 18 | 14 | 23 | 11 | 11 | 11 | 14 | 10 | 12 | 19 | 15 | 17 | 21 | 12 | Present Study |
| South Central India | Madhya Pradesh | Dravidian | PAN57 | 15 | 16 | 12 | 22 | 10 | 11 | 11 | 14 | 9 | 11 | 19 | 16 | 19 | 20 | 12 | Present Study |
| South Central India | Madhya Pradesh | Dravidian | PAN58 | 16 | 16 | 13 | 22 | 11 | 11 | 12 | 14 | 10 | 11 | 18 | 14 | 18 | 20 | 11 | Present Study |
| South Central India | Madhya Pradesh | Dravidian | PAN56 | 16 | 16 | 13 | 22 | 11 | 11 | 12 | 14 | 10 | 11 | 18 | 14 | 18 | 20 | 11 | Present Study |
| South Central India | Madhya Pradesh | Dravidian | PAN62 | 14 | 18 | 14 | 23 | 10 | 12 | 13 | 14 | 10 | 11 | 19 | 16 | 19 | 21 | 12 | Present Study |
| South Central India | Madhya Pradesh | Dravidian | PAN70 | 16 | 16 | 13 | 23 | 10 | 12 | 13 | 14 | 10 | 13 | 18 | 16 | 16 | 21 | 11 | Present Study |
| South Central India | Madhya Pradesh | Dravidian | PAN71 | 16 | 16 | 13 | 23 | 10 | 12 | 13 | 14 | 10 | 13 | 18 | 16 | 16 | 21 | 11 | Present Study |
| South Central India | Madhya Pradesh | Dravidian | PAN72 | 14 | 16 | 14 | 22 | 11 | 13 | 12 | 15 | 10 | 12 | 19 | 15 | 18 | 21 | 11 | Present Study |
| South Central India | Madhya Pradesh | Dravidian | PAN73 | 16 | 16 | 13 | 22 | 11 | 11 | 12 | 14 | 10 | 11 | 18 | 14 | 18 | 20 | 11 | Present Study |
| South Central India | Madhya Pradesh | Dravidian | PAN74 | 15 | 16 | 13 | 23 | 10 | 13 | 13 | 14 | 11 | 13 | 18 | 16 | 16 | 21 | 12 | Present Study |
| South Central India | Madhya Pradesh | Dravidian | PAN75 | 15 | 17 | 13 | 24 | 10 | 11 | 12 | 14 | 9 | 12 | 20 | 15 | 18 | 21 | 11 | Present Study |
| South Central India | Madhya Pradesh | Dravidian | PAN3 | 15 | 16 | 13 | 24 | 11 | 13 | 13 | 15 | 10 | 12 | 18 | 15 | 16 | 21 | 11 | Present Study |
| South Central India | Madhya Pradesh | Dravidian | PAN18 | 15 | 18 | 13 | 24 | 10 | 11 | 11 | 14 | 11 | 10 | 20 | 15 | 16 | 22 | 12 | Present Study |
| South Central India | Madhya Pradesh | Dravidian | PAN39 | 16 | 16 | 14 | 22 | 10 | 11 | 11 | 14 | 10 | 10 | 19 | 16 | 17 | 22 | 13 | Present Study |
| South Central India | Madhya Pradesh | Dravidian | RAJ13 | 15 | 16 | 13 | 24 | 11 | 11 | 13 | 14 | 10 | 12 | 18 | 16 | 17 | 21 | 11 | Present Study |
| South Central India | Madhya Pradesh | Dravidian | RAJ14 | 15 | 16 | 12 | 22 | 10 | 11 | 11 | 14 | 9 | 11 | 19 | 16 | 18 | 20 | 12 | Present Study |
| South Central India | Madhya Pradesh | Dravidian | RAJ17 | 16 | 15 | 13 | 22 | 10 | 11 | 13 | 14 | 11 | 12 | 19 | 15 | 17 | 21 | 11 | Present Study |
| South Central India | Madhya Pradesh | Dravidian | RAJ20 | 15 | 16 | 12 | 22 | 10 | 11 | 11 | 14 | 9 | 11 | 19 | 16 | 18 | 20 | 12 | Present Study |
| South Central India | Madhya Pradesh | Dravidian | RAJ25 | 15 | 18 | 12 | 23 | 10 | 12 | 11 | 14 | 10 | 11 | 19 | 16 | 18 | 20 | 11 | Present Study |
| South Central India | Madhya Pradesh | Dravidian | RAJ29 | 15 | 16 | 12 | 22 | 10 | 11 | 11 | 14 | 9 | 11 | 19 | 15 | 19 | 20 | 12 | Present Study |
| South Central India | Madhya Pradesh | Dravidian | RAJ33 | 15 | 17 | 14 | 24 | 10 | 11 | 12 | 14 | 11 | 10 | 20 | 15 | 16 | 22 | 13 | Present Study |
| South Central India | Madhya Pradesh | Dravidian | RAJ37 | 15 | 18 | 13 | 24 | 10 | 11 | 12 | 14 | 11 | 11 | 20 | 15 | 16 | 22 | 13 | Present Study |
| South Central India | Madhya Pradesh | Dravidian | RAJ39 | 15 | 16 | 12 | 22 | 10 | 11 | 11 | 14 | 9 | 11 | 19 | 16 | 19 | 20 | 11 | Present Study |
| South Central India | Madhya Pradesh | Dravidian | RAJ46 | 13 | 17 | 12 | 21 | 9 | 12 | 11 | 14 | 9 | 11 | 19 | 14 | 16 | 20 | 12 | Present Study |
| South Central India | Madhya Pradesh | Dravidian | RAJ47 | 16 | 17 | 13 | 24 | 10 | 11 | 12 | 14 | 11 | 10 | 20 | 15 | 17 | 22 | 12 | Present Study |
| South Central India | Madhya Pradesh | Dravidian | RAJ51 | 15 | 17 | 13 | 24 | 11 | 11 | 12 | 14 | 10 | 10 | 20 | 15 | 16 | 22 | 12 | Present Study |
| South Central India | Madhya Pradesh | Austroasiatic | Z327 | 15 | 17 | 14 | 21 | 10 | 11 | 14 | 15 | 11 | 11 | 20 | 15 | 17 | 22 | 12 | Present Study |
| South India | Andhra Pradesh | Dravidian | PER46 | 16 | 16 | 13 | 22 | 10 | 11 | 11 | 14 | 9 | 11 | 19 | 16 | 19 | 19 | 12 | Present Study |
| South India | Andhra Pradesh | Dravidian | EDI32 | 14 | 16 | 12 | 22 | 10 | 12 | 11 | 15 | 10 | 12 | 19 | 15 | 16 | 22 | 12 | Present Study |
| South India | Karnataka | Dravidian | S17 | 15 | 17 | 13 | 21 | 10 | 11 | 12 | 14 | 8 | 11 | 19 | 15 | 19 | 21 | 11 | Present Study |
| South India | Karnataka | Dravidian | S23 | 15 | 16 | 14 | 21 | 10 | 13 | 12 | 14 | 8 | 11 | 19 | 15 | 19 | 21 | 11 | Present Study |
| South India | Karnataka | Dravidian | S26 | 15 | 17 | 13 | 21 | 10 | 11 | 12 | 14 | 11 | 11 | 19 | 15 | 19 | 21 | 11 | Present Study |
| South India | Karnataka | Dravidian | S27 | 15 | 17 | 13 | 21 | 10 | 11 | 12 | 14 | 11 | 11 | 19 | 15 | 19 | 21 | 11 | Present Study |
| South India | Karnataka | Dravidian | S32 | 15 | 18 | 14 | 22 | 10 | 13 | 12 | 14 | 9 | 11 | 20 | 15 | 17 | 20 | 12 | Present Study |
| South India | Karnataka | Dravidian | S33 | 15 | 18 | 14 | 22 | 10 | 13 | 12 | 14 | 9 | 11 | 20 | 15 | 17 | 20 | 12 | Present Study |
| South India | Karnataka | Dravidian | S34 | 15 | 17 | 14 | 21 | 10 | 13 | 12 | 14 | 9 | 13 | 19 | 15 | 16 | 20 | 12 | Present Study |
| South India | Karnataka | Dravidian | g42 | 15 | 16 | 13 | 22 | 11 | 11 | 12 | 14 | 9 | 11 | 19 | 16 | 18 | 22 | 11 | Present Study |
| South India | Karnataka | Dravidian | g72 | 15 | 17 | 13 | 21 | 11 | 11 | 13 | 14 | 8 | 11 | 19 | 15 | 19 | 21 | 11 | Present Study |
| South India | Kerala | Dravidian | KUR6 | 15 | 17 | 13 | 24 | 11 | 12 | 11 | 14 | 9 | 11 | 20 | 16 | 18 | 20 | 11 | Present Study |
| South India | Kerala | Dravidian | KUR29 | 15 | 18 | 14 | 22 | 11 | 12 | 11 | 14 | 9 | 11 | 20 | 15 | 18 | 20 | 12 | Present Study |
| South India | Kerala | Dravidian | KUR76 | 15 | 16 | 14 | 22 | 10 | 11 | 12 | 15 | 10 | 12 | 18 | 15 | 16 | 21 | 11 | Present Study |
| South India | Kerala | Dravidian | KUR83 | 15 | 17 | 14 | 22 | 11 | 11 | 11 | 14 | 9 | 11 | 20 | 15 | 18 | 20 | 12 | Present Study |
| South India | Pondicherry | Indo-European | YAD8 | 16 | 17 | 12 | 23 | 10 | 12 | 12 | 14 | 11 | 11 | 20 | 15 | 19 | 21 | 12 | Present Study |
| South India | Pondicherry | Indo-European | YAD14 | 15 | 16 | 14 | 21 | 11 | 12 | 11 | 14 | 10 | 11 | 19 | 16 | 16 | 21 | 11 | Present Study |
| South India | Pondicherry | Indo-European | YAD25 | 15 | 17 | 13 | 24 | 10 | 12 | 12 | 14 | 10 | 11 | 19 | 15 | 16 | 21 | 11 | Present Study |
| South India | Pondicherry | Indo-European | YAD34 | 14 | 15 | 14 | 21 | 11 | 12 | 11 | 15 | 9 | 11 | 19 | 15 | 18 | 22 | 12 | Present Study |
| South India | Pondicherry | Indo-European | YAD48 | 16 | 17 | 13 | 23 | 11 | 11 | 12 | 14 | 11 | 12 | 19 | 15 | 16 | 20 | 12 | Present Study |
| South India | Pondicherry | Indo-European | YAD69 | 16 | 17 | 13 | 23 | 11 | 11 | 12 | 14 | 11 | 12 | 19 | 15 | 17 | 20 | 11 | Present Study |
| South India | Pondicherry | Indo-European | YAD70 | 15 | 16 | 13 | 22 | 10 | 11 | 11 | 14 | 9 | 11 | 19 | 15 | 18 | 21 | 12 | Present Study |
| South India | Pondicherry | Indo-European | YAD72 | 16 | 16 | 13 | 22 | 11 | 12 | 13 | 14 | 10 | 12 | 21 | 16 | 18 | 21 | 12 | Present Study |
| South India | Pondicherry | Indo-European | YAD84 | 16 | 15 | 13 | 23 | 9 | 11 | 13 | 15 | 9 | 12 | 18 | 16 | 18 | 21 | 12 | Present Study |
| South India | Pondicherry | Indo-European | YAD85 | 15 | 17 | 13 | 22 | 10 | 11 | 11 | 14 | 9 | 12 | 18 | 16 | 17 | 20 | 12 | Present Study |
| South India | Pondicherry | Indo-European | YAD86 | 13 | 16 | 12 | 21 | 9 | 11 | 11 | 13 | 10 | 10 | 20 | 15 | 17 | 20 | 13 | Present Study |
| South India | Pondicherry | Indo-European | YAD43 | 16 | 17 | 13 | 23 | 11 | 11 | 12 | 14 | 11 | 11 | 20 | 15 | 16 | 20 | 11 | Present Study |
| South India | Tamil Nadu | Dravidian | NAD55 | 14 | 18 | 13 | 24 | 10 | 11 | 13 | 14 | 10 | 11 | 19 | 16 | 16 | 19 | 12 | Present Study |
| South India | Tamil Nadu | Dravidian | NAD56 | 15 | 16 | 13 | 22 | 10 | 11 | 11 | 13 | 9 | 11 | 19 | 16 | 17 | 20 | 12 | Present Study |
| South India | Tamil Nadu | Dravidian | NAD62 | 15 | 16 | 13 | 22 | 10 | 11 | 11 | 14 | 9 | 11 | 20 | 16 | 17 | 20 | 11 | Present Study |
| South India | Tamil Nadu | Dravidian | NAD66 | 14 | 18 | 13 | 24 | 10 | 11 | 13 | 14 | 10 | 11 | 19 | 16 | 16 | 20 | 12 | Present Study |
| South India | Tamil Nadu | Dravidian | NAD72 | 14 | 18 | 13 | 24 | 9 | 12 | 12 | 14 | 10 | 11 | 20 | 14 | 16 | 21 | 11 | Present Study |
| South India | Tamil Nadu | Dravidian | NAD77 | 15 | 16 | 13 | 23 | 9 | 11 | 12 | 14 | 10 | 12 | 18 | 16 | 18 | 22 | 11 | Present Study |
| South India | Tamil Nadu | Dravidian | NAD82 | 14 | 17 | 13 | 24 | 10 | 11 | 13 | 14 | 10 | 11 | 19 | 16 | 16 | 21 | 12 | Present Study |
| South India | Tamil Nadu | Dravidian | NAD89 | 15 | 16 | 14 | 22 | 10 | 11 | 12 | 14 | 9 | 12 | 19 | 16 | 18 | 21 | 12 | Present Study |
| South India | Tamil Nadu | Dravidian | NAD91 | 13 | 18 | 13 | 22 | 10 | 11 | 11 | 14 | 11 | 11 | 19 | 17 | 18 | 21 | 12 | Present Study |
| South India | Tamil Nadu | Dravidian | NAD98 | 13 | 18 | 13 | 23 | 11 | 11 | 11 | 15 | 11 | 11 | 19 | 15 | 18 | 21 | 11 | Present Study |
| West India | Maharashtra | Indo-European | MK020 | 14 | 16 | 13 | 22 | 10 | 12 | 12 | 14 | 9 | 11 | 19 | 18 | 19 | 21 | 12 | Thangaraj et al. 2010 |
| West India | Maharashtra | Indo-European | MK024 | 15 | 16 | 13 | 21 | 11 | 11 | 13 | 15 | 10 | 13 | 18 | 14 | 16 | 21 | 10 | Thangaraj et al. 2010 |
| West India | Maharashtra | Indo-European | MK026 | 15 | 15 | 13 | 22 | 10 | 11 | 12 | 14 | 9 | 11 | 18 | 15 | 17 | 20 | 12 | Thangaraj et al. 2010 |
| West India | Maharashtra | Indo-European | MK027 | 15 | 17 | 13 | 22 | 10 | 11 | 13 | 14 | 11 | 10 | 20 | 15 | 16 | 21 | 12 | Thangaraj et al. 2010 |
| West India | Maharashtra | Indo-European | MK041 | 15 | 16 | 13 | 22 | 10 | 11 | 12 | 14 | 9 | 11 | 19 | 15 | 17 | 20 | 12 | Thangaraj et al. 2010 |
| West India | Maharashtra | Indo-European | MK047 | 15 | 16 | 14 | 22 | 10 | 11 | 13 | 14 | 11 | 12 | 19 | 18 | 17 | 21 | 13 | Thangaraj et al. 2010 |
| West India | Maharashtra | Indo-European | MK051 | 16 | 16 | 13 | 22 | 10 | 11 | 12 | 14 | 9 | 12 | 19 | 15 | 16 | 20 | 12 | Thangaraj et al. 2010 |
| West India | Maharashtra | Indo-European | MK079 | 15 | 16 | 14 | 22 | 9 | 11 | 12 | 14 | 9 | 11 | 19 | 15 | 18 | 20 | 12 | Thangaraj et al. 2010 |
| West India | Maharashtra | Indo-European | MK083 | 15 | 16 | 14 | 22 | 10 | 11 | 12 | 14 | 9 | 11 | 19 | 17 | 17 | 21 | 13 | Thangaraj et al. 2010 |
| West India | Maharashtra | Indo-European | T014 | 15 | 18 | 13 | 23 | 10 | 13 | 12 | 14 | 9 | 11 | 18 | 15 | 17 | 20 | 12 | Thangaraj et al. 2010 |
| West India | Maharashtra | Indo-European | T037 | 14 | 16 | 14 | 22 | 10 | 11 | 12 | 14 | 9 | 11 | 19 | 16 | 18 | 20 | 12 | Thangaraj et al. 2010 |
| West India | Maharashtra | Indo-European | T046 | 15 | 16 | 14 | 22 | 10 | 11 | 12 | 14 | 9 | 11 | 19 | 15 | 16 | 20 | 12 | Thangaraj et al. 2010 |
| West India | Maharashtra | Indo-European | T069 | 15 | 15 | 14 | 22 | 10 | 11 | 13 | 14 | 9 | 11 | 19 | 15 | 17 | 20 | 12 | Thangaraj et al. 2010 |
