# Supplementary material for: The Phylogeography of Y-Chromosome Haplogroup H1a1a-M82 Reveals the Likely Indian Origin of the European Romani Populations
Source: PLoS One. 2012 Nov 28;7(11):e48477. doi: 10.1371/journal.pone.0048477 (PMC3509117; doi:10.1371/journal.pone.0048477)
Supplement: Table S3 — Regionwise haplogroup frequency in India and Nepal. (DOC) [file pone.0048477.s005.doc]

| **Region** | ***n* (populations)** | ***n* (samples)** | **Frequency** |
| --- | --- | --- | --- |
| Northwest India | 16 | 842 | 0.145 |
| South India | 54 | 1845 | 0.201 |
| Central India | 22 | 863 | 0.148 |
| North India | 23 | 622 | 0.140 |
| East India | 57 | 1706 | 0.084 |
| West India | 15 | 501 | 0.172 |
| Northeast India | 21 | 1090 | 0.001 |
| Andaman Island | 2 | 20 | 0 |
| Terai Nepal | 4 | 197 | 0.107 |
