# Supplementary material for: The Phylogeography of Y-Chromosome Haplogroup H1a1a-M82 Reveals the Likely Indian Origin of the European Romani Populations
Source: PLoS One. 2012 Nov 28;7(11):e48477. doi: 10.1371/journal.pone.0048477 (PMC3509117; doi:10.1371/journal.pone.0048477)
Supplement: Table S4 — The frequency of haplogroup H1a1a-M82 among different world populations. (DOC) [file pone.0048477.s006.doc]

| **Region/Ethnicity** | **Country/Population** | **Size** | **H1a freq. (%)** | **Reference** |
| --- | --- | --- | --- | --- |
| **East/Southeast Asia** |  |  |  |  |
|  | Tibet | 156 | 0 | Gayden et al. 2007 |
|  | Cambodia | 6 | 16.67 | Underhill et al. 2000 |
|  | Laos | 18 | 5.56 | Underhill et al. 2000 |
|  | Japan | 23 | 0 | Sengupta et al. 2006 |
| **North Asia** |  |  |  |  |
|  | Siberia | 18 | 0 | Sengupta et al. 2006 |
| **Middle East and North Africa** |  |  |  |  |
|  | Qatar | 72 | 1.39 | Cadenas et al. 2008 |
|  | United Arab Emirates | 164 | 1.84 | Cadenas et al. 2008 |
|  | Yemen | 62 | 0 | Cadenas et al. 2008 |
|  | Saudi Arabia | 157 | 0.64 | Abu-Amero et al. 2009 |
|  | Oman | 121 | 0 | Abu-Amero et al. 2009 |
|  | Egypt | 147 | 0 | Abu-Amero et al. 2009 |
|  | Somalia | 201 | 0 | Abu-Amero et al. 2009 |
|  | Lebanese | 916 | 0 | Abu-Amero et al. 2009 |
|  | Jordan | 146 | 0 | Abu-Amero et al. 2009 |
|  | Iraq | 203 | 0 | Abu-Amero et al. 2009 |
|  | Turkish | 523 | 0.19 | Cinnioglu et al. 2004 |
|  | Iran | 150 | 2 | Abu-Amero et al. 2009 |
|  | Iran | 938 | 1.2 | Grugni et al. 2012 |
| **Roma-Europe** |  |  |  |  |
|  | Slovakian | 62 | 30.65 | Pamjev et al. 2011 |
|  | Portuguese | 126 | 16.67 | Gusmao et al. 2008 |
|  | Kosovo, Belgrade, Vojvodina | 88 | 43.18 | Regueiro et al. 2011 |
|  | Bulgarian | 248 | 39.52 | Gresham et al. 2001 |
|  | Spanish | 27 | 18.52 | Gresham et al. 2001 |
|  | Croatians | 377 | 20.16 | Battaglia et al. 2009 |
|  | Macedonians | 257 | 13.23 | Peričić et al. 2005 |
|  | Hungarian | 424 | 16.98 | Pamjav et al. 2011 |
|  | Lithuvenian Roma | 20 | 50 | Gresham et al. 2001 |
| **Balkans** |  |  |  |  |
|  | Greeks | 92 | 0 | Battaglia et al. 2009 |
|  | Albanians | 55 | 0 | Battaglia et al. 2009 |
|  | Bosniacs | 324 | 0 | Battaglia et al. 2009 |
|  | Slovenians | 75 | 0 | Battaglia et al. 2009 |
|  | North-East-Italians | 67 | 0 | Battaglia et al. 2009 |
|  | Hungarians | 53 | 0 | Battaglia et al. 2009 |
|  | Czechs | 75 | 0 | Battaglia et al. 2009 |
|  | Poles | 99 | 0 | Battaglia et al. 2009 |
|  | Ukrainians | 92 | 1.1 | Battaglia et al. 2009 |
|  | Herzegovinians | 141 | 0 | Peričić et al. 2005 |
|  | Serbians | 113 | 0.9 | Peričić et al. 2005 |
| **Caucasus** |  |  |  |  |
|  | Caucasians | 1789 | 0 | Yunusbayev et al. 2011 |
|  | Georgians | 66 | 0 | Battaglia et al. 2009 |
|  | Balkarians | 38 | 2.6 | Battaglia et al. 2009 |
| **South Asia** |  |  |  |  |
|  | Nepal | 188 | 4.25 | Gayden et al. 2007 |
|  | Afghanistan | 204 | 3.43 | Haber et al. 2012 |
|  | Malaysian Indians | 301 | 18.94 | Pamjav et al. 2011 |
|  | Terai-Nepal | 197 | 10.66 | Fornarino et al. 2009 |
|  | Hindu New Delhi | 49 | 10.2 | Fornarino et al. 2009 |
|  | Andhra Pradesh Tribals | 29 | 27.6 | Fornarino et al. 2009 |
|  | Northwest India | 842 | 14.49 | Present study |
|  | South India | 1845 | 20.05 | Present study |
|  | Central India | 863 | 14.83 | Present study |
|  | North India | 622 | 13.99 | Present study |
|  | East India | 1706 | 8.44 | Present study |
|  | West India | 501 | 17.17 | Present study |
|  | Northeast India | 1090 | 0.18 | Present study |
|  | Andaman Island | 20 | 0 | Thangaraj et al. 2003 |

Note= For populationwise details about Indian populations please see supplementary Table S1
