# Supplementary material for: The Phylogeography of Y-Chromosome Haplogroup H1a1a-M82 Reveals the Likely Indian Origin of the European Romani Populations
Source: PLoS One. 2012 Nov 28;7(11):e48477. doi: 10.1371/journal.pone.0048477 (PMC3509117; doi:10.1371/journal.pone.0048477)
Supplement: Table S5 — Mean pairwise Fst between different studied groups for haplogroup H1a1a-M82. (DOC) [file pone.0048477.s007.doc]

|  | RP | RC | RS | NWI | WI | NI | NCI | EI | SCI | SI |
| --- | --- | --- | --- | --- | --- | --- | --- | --- | --- | --- |
| Roma Portugal (RP) | 0 |  |  |  |  |  |  |  |  |  |
| Roma Croatia (RC) | 0,3622 | 0 |  |  |  |  |  |  |  |  |
| Roma Serbia (RS) | 0,1580 | 0,4480 | 0 |  |  |  |  |  |  |  |
| Northwest India (NWI) | 0,1707 | 0,3272 | 0,2377 | 0 |  |  |  |  |  |  |
| West India (WI) | 0,1708 | 0,3536 | 0,2704 | 0,0201 | 0 |  |  |  |  |  |
| North India (NI) | 0,3159 | 0,5152 | 0,4059 | 0,0532 | 0,0848 | 0 |  |  |  |  |
| Northcentral India (NCI) | 0,3307 | 0,4855 | 0,3969 | 0,1145 | 0,0998 | 0,0424 | 0 |  |  |  |
| East India (EI) | 0,4646 | 0,6069 | 0,5563 | 0,1385 | 0,1295 | 0,1017 | 0,1125 | 0 |  |  |
| Southcentral India (SCI) | 0,2600 | 0,3919 | 0,3290 | 0,0688 | 0,0473 | 0,0348 | 0,0637 | 0,0878 | 0 |  |
| South India (SI) | 0,2758 | 0,4422 | 0,3334 | 0,0648 | 0,0585 | 0,0218 | 0,0591 | 0,0498 | 0,0241 | 0 |


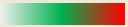


**0 0.61**
