# Supplementary material for: The Phylogeography of Y-Chromosome Haplogroup H1a1a-M82 Reveals the Likely Indian Origin of the European Romani Populations
Source: PLoS One. 2012 Nov 28;7(11):e48477. doi: 10.1371/journal.pone.0048477 (PMC3509117; doi:10.1371/journal.pone.0048477)
Supplement: Table S6 — Modal H1a1a-M82 Y-STR haplotype of different population groups. (DOC) [file pone.0048477.s008.doc]

| **Group** | **DYS19** | **DYS389ab** | **DYS389cd** | **DYS390** | **DYS391** | **DYS392** | **DYS393** | **DYS437** | **DYS438** | **DYS439** | **DYS448** | **DYS456** | **DYS458** | **DYS635** | **H4** |
| --- | --- | --- | --- | --- | --- | --- | --- | --- | --- | --- | --- | --- | --- | --- | --- |
| Roma-Portugal | 15 | 16 | 14 | 22 | 10 | 11 | 12 | 14 | 9 | 11 | 19 | 15 | 17 | 20 | 12 |
| Roma-Serbia | 15 | 16 | 14 | 22 | 10 | 11 | 12 | 14 | 9 | 11 | 19 | 15 | 18 | 20 | 12 |
| Roma-Croatia | 15 | 16 | 14 | 22 | 10 | 11 | 12 | 14 | 9 | 11 | 18 | 15 | 17 | 20 | 12 |
| Roma all | 15 | 16 | 14 | 22 | 10 | 11 | 12 | 14 | 9 | 11 | 19 | 15 | 17 | 20 | 12 |
| Northwest India | 15 | 16 | 14 | 22 | 10 | 11 | 12 | 14 | 9 | 11 | 19 | 15 | 17 | 20 | 12 |
| West India | 15 | 16 | 13 | 22 | 10 | 11 | 12 | 14 | 9 | 11 | 19 | 15 | 17 | 20 | 12 |
| North India | 15 | 16 | 13 | 22 | 10 | 11 | 11 | 14 | 10 | 11 | 19 | 15 | 17 | 21 | 12 |
| East India | 15 | 16 | 13 | 22 | 10 | 11 | 13 | 14 | 10 | 11 | 18 | 15 | 16 | 21 | 11 |
| Northcentral India | 16 | 16 | 13 | 22 | 10 | 11 | 11 | 14 | 10 | 11 | 19 | 15 | 16 | 21 | 12 |
| Southcentral India | 15 | 16 | 13 | 22 | 10 | 11 | 12 | 14 | 9 | 11 | 20 | 16 | 17 | 20 | 12 |
| South India | 15 | 17 | 13 | 22 | 10 | 11 | 12 | 14 | 9 | 11 | 19 | 15 | 18 | 21 | 12 |
| Afghani | 15 | 16 | 13 | 24 | 10 | 11 | 12 | 14 | 9 | 11 | 18 | 15 | 16 | 21 | 11 |
