# Supplementary material for: The Phylogeography of Y-Chromosome Haplogroup H1a1a-M82 Reveals the Likely Indian Origin of the European Romani Populations
Source: PLoS One. 2012 Nov 28;7(11):e48477. doi: 10.1371/journal.pone.0048477 (PMC3509117; doi:10.1371/journal.pone.0048477)
Supplement: Table S7 — Average mutational distances from Roma Modal haplotype. (DOC) [file pone.0048477.s009.doc]

| **Group** | **Distance** |
| --- | --- |
| Roma-Portugal | 1.14 |
| Roma-Serbia | 1.66 |
| Roma-Croatia | 1.37 |
| Roma all | 1.37 |
| Northwest India | 5.86 |
| West India | 5.23 |
| North India | 8.04 |
| East India | 9.14 |
| Northcentral India | 7.94 |
| Southcentral India | 8.84 |
| South India | 8.78 |
| Afghani | 7.83 |
